# Unfolding the Geometric Structure and Multiple Timescales of the Urea–Urease pH Oscillator

**DOI:** 10.1007/s00332-026-10336-x

**Published:** 2026-11-05

**Authors:** Arthur V. Straube, Guillermo Olicón Méndez, Stefanie Winkelmann, Felix Höfling, Maximilian Engel

**Affiliations:** 1 Zuse Institute Berlin, Takustraße 7, 14195 Berlin, Germany; 2 Department of Mathematics and Computer Science, Freie Universität Berlin, Arnimallee 6, 14195 Berlin, Germany; 3 Institut für Mathematik, Universität Potsdam, Karl-Liebknecht-Straße 24, 14476 Potsdam, Germany; 4 KdVI Institute for Mathematics, University of Amsterdam, Amsterdam, The Netherlands

**Keywords:** Fast-slow system, Geometric singular perturbation theory, Relaxation oscillations, Biochemical oscillators, Clock reactions, 34E15, 34C26, 92C45

## Abstract

We study a two-variable dynamical system modeling pH oscillations in the urea–urease reaction within giant lipid vesicles — a problem that intrinsically contains multiple, well-separated timescales. Building on an existing, deterministic formulation via ordinary differential equations, we resolve different orders of magnitude within a small parameter and analyze the system’s limit cycle behavior using geometric singular perturbation theory (GSPT). By introducing two different coordinate scalings — each valid in a distinct region of the phase space — we resolve the local dynamics near critical fold points, using the extension of GSPT through such singular points due to Krupa and Szmolyan. This framework enables a geometric decomposition of the periodic orbits into slow and fast segments, which allows us to obtain a closed-form estimate for the oscillation period. Moreover, we link the existence of pH oscillations to an underlying biochemical asymmetry, namely, the differential transport across the vesicle membrane.

## Introduction

Oscillatory rhythms are ubiquitous in biophysical systems, underpinning essential functions such as spatiotemporal self-organization, cellular signaling, metabolism, and homeostasis (Epstein 1991; Goldbeter 1996; Epstein and Pojman 1998; Novák and Tyson 2008; De Kepper et al. 2009; Yashin et al. 2012; Göth and Dzubiella 2025). These dynamics are often mediated by enzymes, which play a central role in intracellular kinetics and typically operate on multiple timescales, embedded in complex feedback mechanisms that regulate their activity (Goldbeter 1993; Barkai and Leibler 1997; Cornish-Bowden 2004; Alon 2007; Rubin and Sollich 2016; Milster et al. 2023). Enzymatic activity and cellular function are highly sensitive to environmental factors, in particular to the level of acidity or pH (Alberty and Massey 1954; Cornish-Bowden 2004; Fidaleo and Lavecchia 2003; Casey et al. 2010; Yashin et al. 2012). Systems exhibiting periodic changes in pH are known as *pH oscillators* (Rábai 1998; McIlwaine et al. 2006; Orbán et al. 2015; Horváth et al. 2018; Dúzs et al. 2023). While many studies of such oscillators have focused on closed reactors, recent work has increasingly turned to open reaction compartments that exchange matter with their surroundings (Miele et al. 2016, 2018; Muzika et al. 2019; Straube et al. 2021; Miele et al. 2022; Leathard et al. 2023; Ridgway-Brown et al. 2025; Itatani et al. 2025; Okano and Yoshimoto 2025), enabling the sustained operation of chemical switches or clocks (Lente et al. 2007; Hu et al. 2010; Bubanja et al. 2018). In this work, we analyze the urea–urease reaction confined to a lipid vesicle—a minimal model system in which urease catalyzes the hydrolysis of urea in a pH-dependent manner, while the vesicle membrane permits exchange of acid and substrate with the surrounding medium. Such a setting has relevance in biotechnology, e.g., for pH photo switches (Ivanov et al. 2025), pH-controlled polymerization (Mai et al. 2021; Mahmud and Pojman 2025) and gelation processes (Bashir et al. 2024), chemical oscillators in open reactors (Galanics and Szalai 2024), and spatiotemporal gating in signal transduction (Mahato et al. 2025). The chemical set-up gives rise to a fast–slow oscillatory dynamics that can be captured by low-dimensional models (Bánsági and Taylor 2014; Straube et al. 2023). Controlling the timescale separation, we uncover the geometric structure underlying the resulting limit cycle dynamics using methods from geometric singular perturbation theory (GSPT) (Kuehn 2015; Wechselberger 2020) which has proven to be a powerful mathematical tool in the context of (bio)chemical reactions (Engel and Méndez 2026; Gucwa and Szmolyan 2009; Kosiuk and Szmolyan 2011; Kuehn and Szmolyan 2015).

**Fig. 1:**
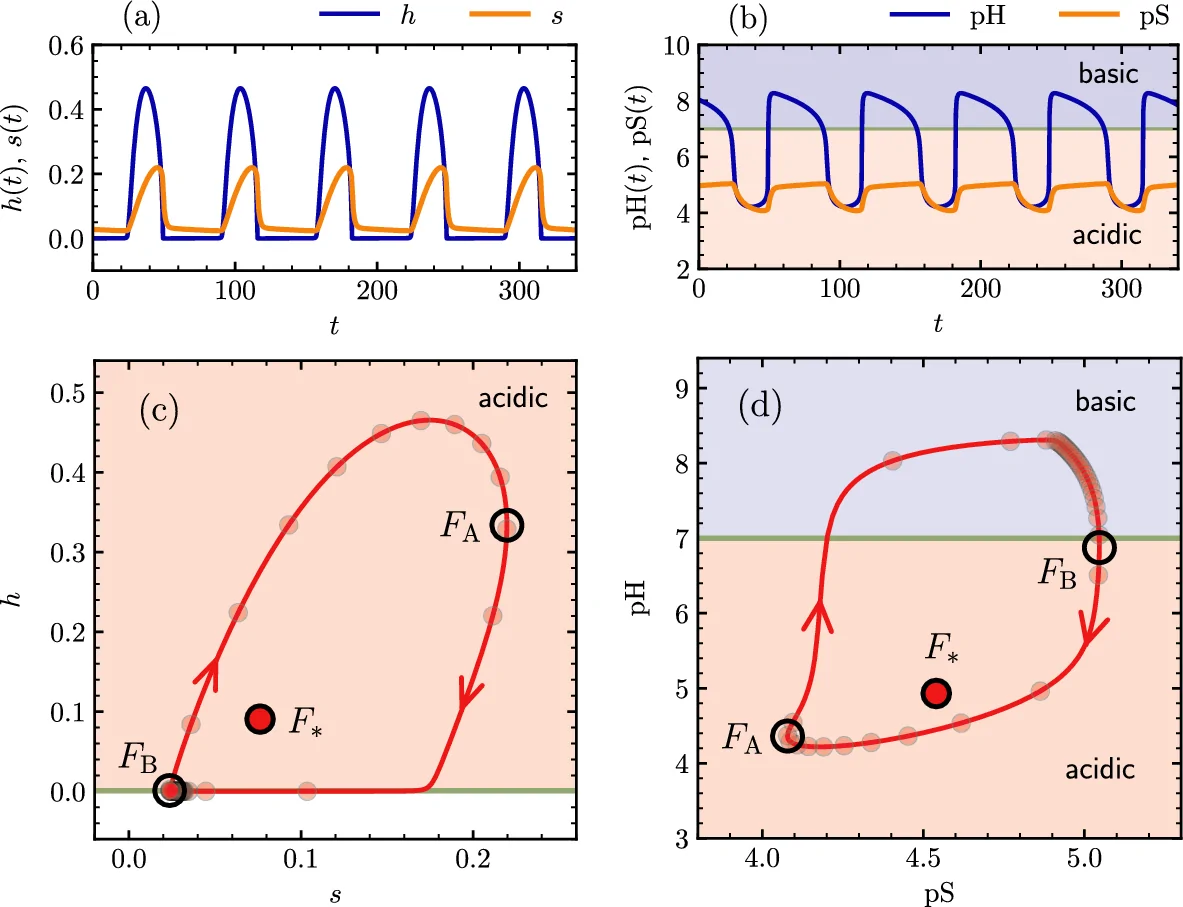
Panels **a** and **b** show a typical numerical solution of Eq. (1) for the parameter values listed in Table 3; panels **c** and **d** depict the corresponding limit cycles for the same data (red solid lines). Left panels [**a** and **c**] use the original (*s*, *h*) variables, while right panels [**b** and **d**] employ the negative logarithms $(\hbox {pS},\hbox {pH})$, commonly used in chemistry (Straube et al. 2021, 2023), see Eq. (A10). In the bottom panels, arrows indicate the direction of motion, and disks show the evolution of the variables sampled equidistantly in time. The limit cycle encloses the only fixed point (dark red disc in panels **c** and **d**), which is repelling and located at $F_*=(s_*,h_*)\approx (0.0762, 0.0906)$ and $(\hbox {pS}_*,\hbox {pH}_*)\approx (4.54, 4.93)$, respectively. The turning points $F_\text {A}$ and $F_\text {B}$ mark those points on the limit cycle with the largest and smallest values of *s* and the smallest and largest values of $\hbox {pS}$, respectively. Note that smaller values of pS and pH correspond to larger values of *s* and *h* and vice versa, which effectively flips the direction of the axes and changes the relative location of points $F_\text {A}$ and $F_\text {B}$. The horizontal green line represents the neutral value, $\hbox {pH} = 7$ ($h=h_\text {max}$, see Sect. 2.1), separating acidic ($\hbox {pH} < 7$, $h > h_\text {max}$, shaded light red) and basic ($\hbox {pH} > 7$, $h < h_\text {max}$, shaded light blue in panels **b** and **d**) domains (Color figure online)

*The model.* We study the two-variable ODE model derived in  (Straube et al. 2023), which deterministically describes pH oscillations in the urea–urease reaction confined to a giant lipid vesicle. Further reduction of this model (Appendix A) leads to the following dynamical system in the dimensionless variables *s* ? and h⩾0, representing rescaled concentrations of the substrate molecules S and hydrogen ions ${\hbox {H}^+}$, respectively:

1a $$\begin{aligned} \frac{ds}{dt}&= f(s,h) := -r(h) s + K_s\,, \end{aligned}$$

1b $$\begin{aligned} \frac{d h}{dt}&= g(s, h) := - q(s,h) + K_h(1-h)\,, \end{aligned}$$

where $K_s$, $K_h>0$ are positive constants. The rate function *r*(*h*), which determines the consumption of the substrate *s*, has the form

2 $$\begin{aligned} r(h) = \bigl (\beta \varepsilon _1/h + 1 + \beta h/\varepsilon _1 \bigr )^{-1} >0, \end{aligned}$$

with a small parameter $\varepsilon _1 > 0$ and some constant β>0. This function exhibits a single maximum and tends to zero as $h\rightarrow \infty$ or h→0. The consumption of hydrogen h⩾0 is governed by the reaction speed *q*(*s*, *h*), which is given as the non-negative root of the quadratic equation $q^2 + v(h) q - K\varepsilon _2^{-1} r(h) h^2 s = 0$:

3 $$\begin{aligned} q(s,h) = - \frac{1}{2} v(h) + \frac{1}{2}\sqrt{v(h)^2+4 K \varepsilon _2^{-1} r(h) h^2 s} \geqslant 0 \,, \end{aligned}$$

where *v*(*h*) is defined as

4 $$\begin{aligned} v(h)&:= \alpha K \varepsilon _2^{-1} h^2 -K_h(1-h) \, , \end{aligned}$$

with constants $\varepsilon _2$, α, *K*, $K_h >0$. We note that for fixed *s*, the function *q*(*s*, *h*) vanishes as $h\rightarrow \infty$, while as h→0 it asymptotically behaves like -v(h). Second, $\varepsilon _1$ appears only in the combination $h/\varepsilon _1$ and $\varepsilon _2$ only in $h^2/\varepsilon _2$; this observation has been motivating for the approach followed below. An exemplary numerical solution of the ODE system (1) is plotted in Fig. 1a.

*Biochemical background and interpretation.* System (1) models the urease-assisted catalysis of urea, serving as the substrate S, with the reaction rate depending on the hydrogen ion (or proton) concentration, i.e., on the pH value quantifying the acidity. Urease, an enzyme acting as a biological catalyst, regulates the speed of the catalytic reaction via the term *r*(*h*)*s*, where *r*(*h*) is the hydrogen-dependent reaction rate. The effective decay of hydrogen (Bánsági and Taylor 2014; Straube et al. 2023) is governed by the function *q*(*s*, *h*), which captures the nonlinear coupling between substrate and proton concentrations.

The described process occurs in an open reactor – a unilamellar vesicle enclosed by a membrane partially permeable to chemical species. Exchange with the surrounding medium ensures a continuous supply of both substrate and acid, with influx rate constants $K_s$ and $K_h$, respectively. Inside the vesicle, the core reaction acts as a chemical switch or clock (Lente et al. 2007; Hu et al. 2010; Bubanja et al. 2018), rapidly driving the system from acidic to basic conditions. In turn, the external inflow of acid and substrate resets the pH clock after each cycle, enabling sustained oscillations. These oscillations result from the interplay between internal switching dynamics and external replenishment, and are characterized by fast–slow transitions between acidic and basic phases. A detailed derivation of the simplified, dimensionless system (1) from a previously reduced model of the urea–urease reaction network (Straube et al. 2023) is presented in Appendix A.

Previous studies have shown that the system under consideration—like those in Refs. Bánsági and Taylor (2014); Straube et al. (2023)—captures a practically relevant and experimentally realistic setting; for further insights, see Acar et al. (2025). One of the strengths of our study is to build on this foundation and rigorously analyze the model’s complex oscillatory behavior. However, due to this realism and unlike many mathematically idealized systems, the present model does not feature a single small parameter that would directly justify a standard fast–slow decomposition or suggest an obvious timescale separation. For parameter regimes relevant to experiments, numerical integration of system (1) reveals stable limit cycle solutions, characterized by very different time scales along the segments of the cycle (Fig. 1). This timescale separation manifests itself in the form of relaxation oscillations, which fall outside the direct applicability of classical singular perturbation theory. In particular, the dynamics is governed by multiple small dimensionless parameter combinations, none of which alone controls the system’s behavior.

The entanglement of multiple small parameters forms an obstacle to a straightforward application of GSPT, which we overcome by identifying a suitable formalization of the timescale separation. This enables the application of GSPT, combining the conventional Fenichel’s theory for normally hyperbolic critical manifolds (Fenichel 1979) with its extension to nonhyperbolic points, such as folds and canards, developed via blow-up techniques (Krupa and Szmolyan 2001). Using this framework, we characterize the structure of the limit cycle and quantify its decomposition into alternating fast and slow segments. Two key components of the limit cycle are stable branches of distinct critical manifolds—one under acidic pH conditions, the other under basic pH conditions—each requiring a different rescaling to resolve the local dynamics. We complete the global picture of the oscillation mechanism by constructing transition maps between suitable Poincaré sections.

In Sect. 2, we present initial observations, establish the existence of a limit cycle, and introduce a rescaling in terms of a single small parameter. The existence of a limit cycle was conjectured in earlier work (Straube et al. 2023), based on the presence of a single repelling fixed point and an application of the Poincaré–Hopf index theory. Here, we provide a more rigorous proof using the Poincaré–Bendixson theorem. Section 3 contains the core of our analysis: we apply GSPT to resolve the dynamics near the fold points at low and neutral pH (Sects 3.1 and 3.2), construct the global structure of the limit cycle and compare with numerical solutions (Sect. 3.3). We discuss the implications for the chemical system at hand (Sect. 4) and conclude in Sect. 5.

## Initial Observations and Analytical Setup

In this section, we briefly discuss a parameter regime where Eq. (1) exhibits oscillatory solutions and we give a necessary conditions for the existence of a limit cycle. We suggest a coupling of the small parameters $\varepsilon _1$ and $\varepsilon _2$ to proceed with a single small parameter ε, which allows converting system (1) into a fast-slow system in standard form.

### Observation of Sustained Oscillations

Figure 1 shows a typical limit cycle solution of system (1), using the variables (*s*, *h*) and, alternatively, their negative logarithms, $(\hbox {pS},\hbox {pH})$, employed in biochemistry (see Eq. (A10) for the conversion relation). From the representations in Fig. 1, one anticipates the presence of multiple timescales: in panels (a) and (b), kinks and rapid variations in time and, in panels (c) and (d), an uneven spacing of markers placed equidistantly in time along the solution, especially in the vicinity of point $F_\text {B}$. The chosen parameter values are listed in Table 3 in Appendix A and are the same as in a previous study (Straube et al. 2023), approximately resembling the experimental situation: while β is fixed by the properties of the urease enzyme and $K_s$ and $K_h$ are given by the specific permeabilities of the vesicle membrane, α, $\varepsilon _1$, and $\varepsilon _2$ can be controlled through the external concentrations of substrate and hydrogen ion, outside of the vesicle.

The position $h_\text {max}$ of the maximum of the consumption rate *r*(*h*) (see Eq. (2)) is determined by $r'(h_\text {max})=0$, yielding $h_{\text {max}}=\varepsilon _1$. For the given parameters, it corresponds to the neutral value of $\hbox {pH}=7$ (green horizontal lines in Fig. 1b, d). At $h=h_{\text {max}}$, the conversion of substrate into product is most efficient and any deviation from the optimal level of $\hbox {pH}$ slows down the corresponding reaction rate *r*(*h*). The limit cycle crosses this line and one part of it belongs to the acidic domain (shaded light red) with $\hbox {pH} < 7$ ($h>h_{\text {max}}$) and part to the basic domain (shaded light blue), where $\hbox {pH} > 7$ ($h<h_{\text {max}}$). Using linear scales for the variables *s* and *h*, as in Fig. 1a and c, the basic region is not well resolved: the neutral value, $\hbox {pH} = 7$, corresponds to $h=h_{\text {max}} \approx {7.7 \times 10^{-4}}$), being compressed and pushed against the h=0 axis. Accordingly, the turning point $F_\text {A}$ lies deeply in the acidic domain, while the turning point $F_\text {B}$ is located in the vicinity of the neutral pH. As we will show below, the position of the turning point $F_\text {B}$, which underlies the geometric structure of the limit cycle, is determined exactly by the maximum of the function *r*(*h*).

### Existence of the Limit Cycle

The dynamical system under consideration, Eq. (1), at long times, approaches either a steady-state solution or oscillations (Straube et al. 2023). Focusing on the limit cycle, we briefly outline the region of parameters where sustained oscillations take place.

The *s*- and *h*-nullclines of system (1), denoted as $\mathcal {N}_s$ and $\mathcal {N}_h$, respectively, are parametrized as

5a $$\begin{aligned} \mathcal {N}_s = \{ (n_s(h), h): h \geqslant 0 \}, &  n_s(h)&:= \frac{K_s}{r(h)}\,, \end{aligned}$$

and

5b $$\begin{aligned} \mathcal {N}_h = \{ (n_h(h), h): h \geqslant 0 \}, &  n_h(h)&:= \frac{\alpha K_h(1-h)}{r(h)} \,. \end{aligned}$$

The unique equilibrium $F_*=(s_*,h_*) \in \mathbb {R}^2_{>0}$ solves $n_h(h_*)=n_s(h_*)=:s_*$ for

6 $$\begin{aligned} s_* = \frac{K_s}{r(h_*)}> 0, \qquad h_* = 1 - \frac{K_s}{\alpha K_h} > 0\,. \end{aligned}$$

For the parameter values in Table 3, used to generate data in Fig. 1, we obtain $F_*=(s_*,h_*)\approx (0.0762, 0.0906)$ or $(\hbox {pS}_*,\hbox {pH}_*)\approx (4.54, 4.93)$.

#### Remark 1

In the following, we require that $\alpha K_h > K_s$, so that $F_*$ exists in the positive quadrant, in particular, the concentration $h_*$ is positive. We note that this condition on the parameters aligns with a general claim made in a previous study for the existence of a limit cycle—namely, differential transport of substrate and hydrogen across the membrane (Bánsági and Taylor 2014), which will be discussed further in Sect. 5. The ratio $K_h/K_s$ quantifies the difference in the transport of the hydrogen ions and substrate across the membrane, and $(2\alpha )^{-1}=[\hbox {S}_\text {ext}]/[\hbox {H+}_\text {ext}]$ represents the ratio of their external concentrations, see Appendix A.

**Fig. 2:**
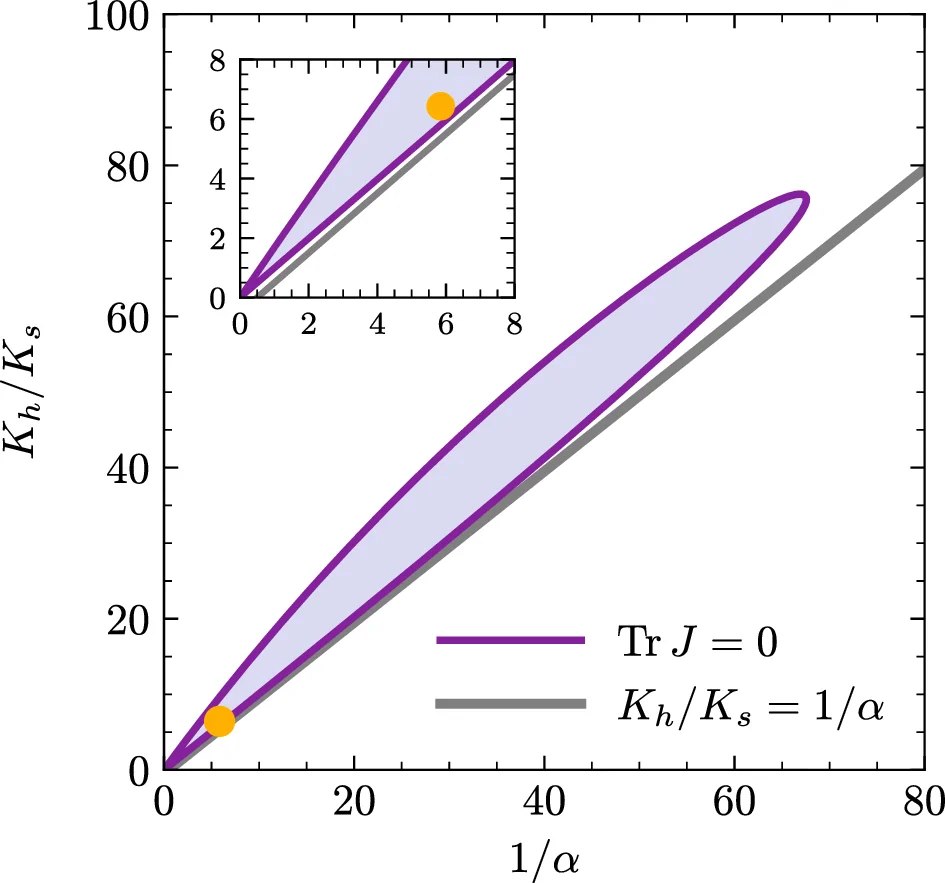
Numerical results for the domain of oscillation in the parameter combinations $(K_h/K_s, \alpha ^{-1})$ at fixed values of β, $\varepsilon _1$, and $\varepsilon _2$ (Table 3). The curve $\textrm{Tr}\, J = 0$ (violet line) separates the regions of stable (attractive) and unstable (repelling) behavior of the fixed point. Oscillations occur in the blue-shaded region, where the fixed point is a repelling node or focus, $\textrm{Tr}\, J > 0$. The yellow disc marks the parameter values of the real-world system (Bánsági and Taylor 2014; Straube et al. 2023) (see Tables 2 and 3), for which the limit cycle is shown in Fig. 1; the corresponding point lies within the region of oscillation, close to the lower boundary (see inset). A lower bound for oscillations is given by the straight line, $K_h/K_s = \alpha ^{-1}$, see Eq. (6) (Color figure online)

The stability of the equilibrium point is determined by the Jacobian matrix of Eq. (1) at $F_*$, which we denote as $J=\partial (f,g)/\partial (s,h)\vert _{F_*}$. The trace $\textrm{Tr}\, J$ exhibits a sign change, suggesting that the system admits a *Hopf bifurcation* (Kuznetsov 2004). Figure 2 depicts the numerically determined domain of oscillations in the parameter plane $(K_h/K_s, 1/\alpha )$. We computed the boundary of the oscillatory domain (violet curve) by numerical continuation of the algebraic equation $\textrm{Tr}\,J=0$, using the continuation routine PITCON (Rheinboldt and Burkardt 1983, 1991); detailed formulas can be found in Appendix A of Straube et al. (2023). Outside this domain, the orbits of the system converge to the unique steady state, provided $\alpha K_h>K_s$. The domain of oscillations in the parameter space is bounded from below by the condition $K_h/K_s = \alpha ^{-1}$.

**Fig. 3:**
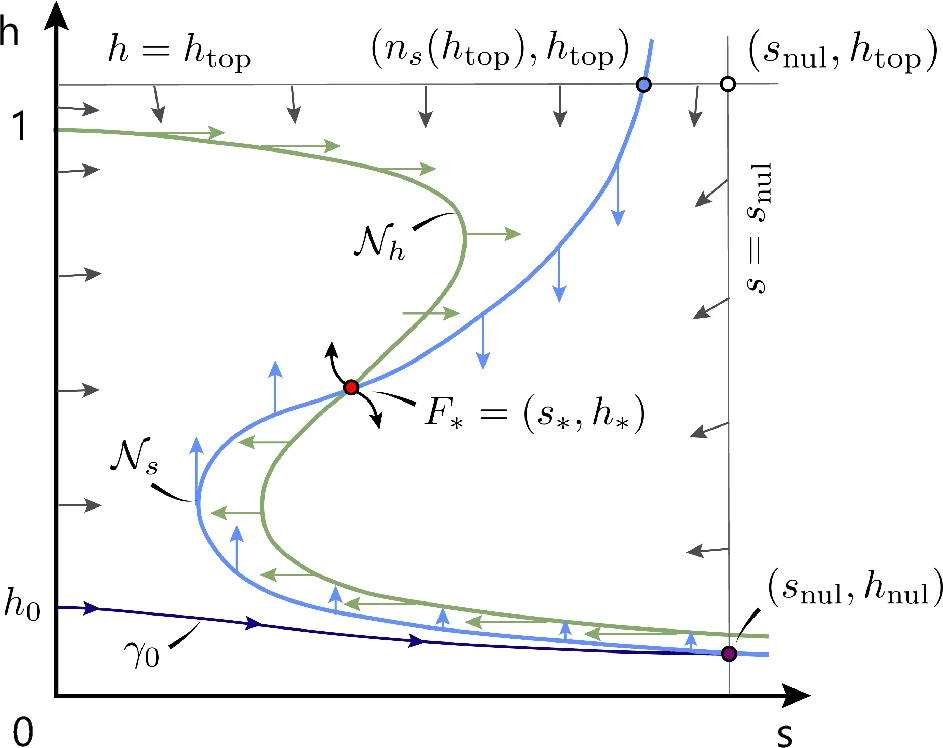
A schematic supporting the proof of Prop. 1. The *s*- and *h*-nullclines, $\mathcal {N}_s$ and $\mathcal {N}_h$, are shown as light blue and green lines, respectively; arrows indicate the direction of the flow field at the corresponding nullclines. The top, $h=h_\text {top}$, and right, $s=s_\text {nul}$, border of the compact region are given by the horizontal and vertical straight lines, respectively. The bottom border is given by the semiorbit $\gamma _0$ starting at $(0,h_0)$ and ending at $(s_\text {nul}, h_\text {nul})$ and is plotted in dark blue. Other characteristic intersection points, including the fixed point $F_*=(s_*,h_*)$, are shown as colored circle markers (Color figure online)

By means of standard phase-plane analysis, the existence of an attracting limit cycle of (1) is guaranteed as long as the equilibrium $F_*$ is unstable, as stated in the following proposition:

#### Proposition 1

Consider system (1) with $\alpha K_h>K_s$ such that $F_*$ given by Eq. (6) is the equilibrium. If the parameter values are such that $\text {Tr}\, J\vert _{F_*}$ and $\text {Det}\, J\vert _{F_*}$ are positive, then system (1) admits an attracting limit cycle contained in the positive quadrant $\mathbb {R}^2_{\geqslant 0}:= \{(s,h):s,\,h\geqslant 0\}$.

#### Proof

We construct a positively invariant compact region $\mathcal {R}$ contained in $\mathbb {R}^2_{>0}$. First, let us fix any $h_\text {top}>1$, a sufficiently large $s_\text {nul}>\max \left\{ K_s/r(h_\text {top}), s_*\right\}$, and the uniquely determined point $(s_\text {nul},h_\text {nul})\in \mathcal {N}_s$ (see Fig. 3). Choose the unique initial condition $(0,h_0)$ such that its forward semiorbit $\gamma _0:=\{(s(t),h(t)): t\geqslant 0, \;(s(0),h(0))=(0,h_0)\}$ intersects $\mathcal {N}_s$ at $(s_\text {nul},h_\text {nul})$. Consider the compact region $\mathcal {R}\subset \mathbb {R}^2_{>0}$ delimited by $\gamma _0$, $\{s=s_\text {nul}\}$, $\{h=h_\text {top}\}$, and $\{ s=0\}$, which is positively invariant for sufficiently large $s_\text {nul}$, see Fig. 3. Indeed, the vector field given by Eq. (1) points inwards on $\partial \mathcal {R}$, apart from $\gamma _0$ on which it is tangent. This follows from the facts that

7a $$\begin{aligned} \begin{aligned}&\dot{s} > 0, &  \text { when } s=0, \end{aligned} \end{aligned}$$

7b $$\begin{aligned} \begin{aligned}&\dot{s} < 0, &  \text { when } s=s_\text {nul} \text { and } h\in (h_\text {nul},h_\text {top}], \end{aligned} \end{aligned}$$

7c $$\begin{aligned} \begin{aligned}&\dot{h} \leqslant K_h(1-h) < 0, &  \text { when } h=h_\text {top}>1 . \end{aligned} \end{aligned}$$

The second line, for $s=s_\text {nul}$, follows from noting that $s_\text {nul} = K_s / r(h_\text {nul}) > K_s / r(h_\text {top})$ and, hence, $s_\text {nul} > K_s / r(h)$ for all $h\in (h_\text {nul},h_\text {top}]$, using that *r*(*h*) is continuous such that $K_s - r(h)s_\text {nul}$ can only change sign at $h_\text {nul}$. The result follows from the Poincaré–Bendixson theorem (cf. e.g.  Coddington and Levinson (1955) [Chapter 16, Theorem 2.1]) since $F_*$ is an unstable node or focus, as indicated by $\text {Tr}\, J\vert _{F_*}>0$ and $\text {Det}\, J\vert _{F_*}>0$. □

### Introduction of a Single Small Parameter

We have seen that, for a suitable choice of parameter values, system (1) admits an attracting limit cycle, which displays slow and fast regimes. We will demonstrate below that the time scale separation emerges in a suitable limit process for the small parameters $\varepsilon _1$ and $\varepsilon _2$. While it is difficult to associate a specific chemical interpretation to these parameter combinations, one observes that $\varepsilon _1^2$ and $\varepsilon _2$ are of similar order of magnitude (see Table 3, and by the only occurrence of $\varepsilon _1$ and $\varepsilon _2$ through the combinations $h/\varepsilon _1$ and $h^2/\varepsilon _2$ in the model, Eqs. (2)–(4)). This structure suggests to use a scaling $\varepsilon _1=\mathcal {O}(\varepsilon )$ and $\varepsilon _2=\mathcal {O}(\varepsilon ^2)$ as $\varepsilon \rightarrow 0$ such that $h/\varepsilon _1$ and $h^2/\varepsilon _2$ can remain balanced if $h = \mathcal {O}(\varepsilon )$. Concretely, we set

8 $$\begin{aligned} \varepsilon _1 =: C \varepsilon \, \quad \text {and} \quad \varepsilon _2 =: A^{-1} \varepsilon ^{2}\,, \end{aligned}$$

with the constant C≈0.77 chosen for convenience such that $\varepsilon = 10^{-3}$ for the parameters of the real-world system in Table 3; it follows that A≈1.4. Upon substituting in Eq. (1), the system reads

9a $$\begin{aligned} \frac{ds}{dt}&= -r_\varepsilon (h) s + K_s\,, \end{aligned}$$

9b $$\begin{aligned} \frac{d h}{dt}&= - q_\varepsilon (s,h) + K_h(1-h)\,, \end{aligned}$$

with the functions

10a $$\begin{aligned} r_\varepsilon (h)&:= \frac{1}{\beta C (h/\varepsilon )^{-1} + 1 + \beta C^{-1} (h/\varepsilon )}, \end{aligned}$$

10b $$\begin{aligned} q_\varepsilon (s, h)&:= \frac{1}{2}\sqrt{v_\varepsilon (h)^2+ 4 A K r_{\varepsilon }(h)(h/\varepsilon )^2 s}- \frac{1}{2} v_\varepsilon (h), \end{aligned}$$

where $v_\varepsilon (h)$ is the quadratic function

11 $$\begin{aligned} v_\varepsilon (h)&:= \alpha A K (h/\varepsilon )^2 -K_h(1-h) \,, \end{aligned}$$

as induced by *v*(*h*) defined in Eq. (4). The equilibrium of Eq. (9) is calculated analogously to Eq. (6) and is located at $F_{*,\varepsilon }:= (s_{*,\varepsilon }, h_{*,\varepsilon }) = (K_s/r_\varepsilon (h_*), h_*)$; in particular, $h_{*,\varepsilon }=h_*$ is independent of ε, but $s_{*,\varepsilon } = \mathcal {O}(\varepsilon ^{-1})$ diverges as $\varepsilon \rightarrow 0$.

The ε-dependent system (9) has been obtained by replacing two small parameters with a single parameter ε, via relation (8). We note that finding such a polynomial expression for common scalings constitutes a part of the *process-oriented GSPT* procedure, which has been put forward in the context of calcium dynamics (Jelbart et al. 2022).

## Fast-Slow Analysis of the System

**Fig. 4:**
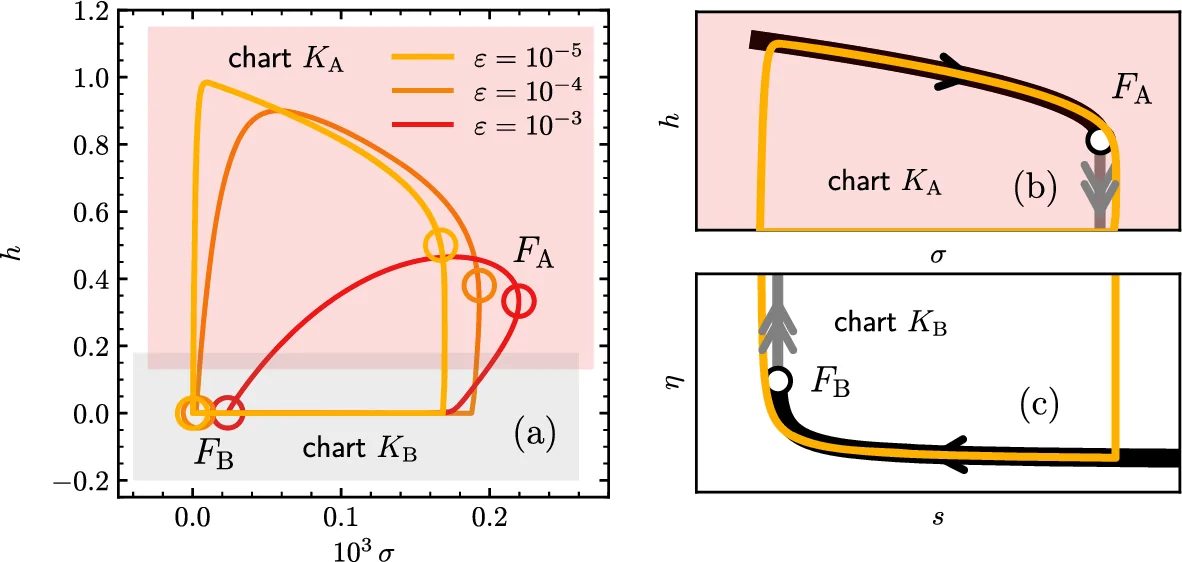
**a**: Numerical solutions of Eq. (9) for $\varepsilon = 10^{-5}, 10^{-4}, 10^{-3}$ showing the limit cycle trajectories and their fold points in the variables (σ,h) with $\sigma = \varepsilon s$. Shaded regions depict the mutual arrangement of the charts $K_\text {A}$ and $K_\text {B}$ associated with the two rescalings, as described in the text. **b** and **c**: Charts $K_\text {A}$ in the variables (σ,h) and $K_\text {B}$ in the variables (s,η), showing the transition from their slow manifolds (solid black lines) to fast fibers (solid gray lines) at the fold points $F_\text {A}$ and $F_\text {B}$ (open circles) for ε=0, respectively; the variables are connected through $\sigma =\varepsilon s$ and $h = \varepsilon \eta$. The yellow solid line is an ε-perturbed trajectory ($\varepsilon =10^{-5}$), which illustrates how switching between the charts leads to a closed orbit (Color figure online)

For the fast-slow analysis of the pH oscillator, described by the dynamical system (9), we employ GSPT techniques (Kuehn 2015; Wechselberger 2020) to obtain and characterize the critical manifold, which pertains to the singular limit (ε=0) and reveals the geometric structure underlying the limit cycle for ε small enough. Figure 1 illustrates the limit cycle for the original system, which, after rescaling of constants, corresponds to $\varepsilon = 10^{-3}$; decreasing ε further enhances the timescale separation. Numerical integration of Eq. (9) shows that, while the limit cycle deforms, it remains bounded in the *h*-variable, but progressively extends in the *s*-variable. However, when represented in terms of the rescaled variable $\sigma = \varepsilon s$, see Fig. 4a, the limit cycle becomes bounded and appears to converge to a specific shape as ε is decreased. This observation motivates us, in Sect. 3.1, to perform the fast-slow analysis in the variables (σ,h). It reveals the slow and fast motions in the acidic domain ($h > h_\text {max}$), including the fold point $F_\text {A}$, but it cannot resolve the fold point $F_\text {B}$ approached from the basic domain ($h < h_\text {max}$), which is close to the h=0 line. We address this issue in Sect. 3.2 by introducing another rescaling, (s,η) with $\eta = h/\varepsilon$, which can capture the dynamics near $F_\text {B}$, but not in the acidic regime.

The two rescalings provide different perspectives on the limit cycle, corresponding to two charts $K_\text {A}$ and $K_\text {B}$, which overlap and can be matched, as depicted in Fig. 4. In both charts, we first focus on the singular limit ε=0 and identify the critical manifolds, which are *normally hyperbolic* away from the points $F_\text {A}$ and $F_\text {B}$. These invariant manifolds persist as invariant slow manifolds for ε small enough, and can be extended beyond the generic fold points $F_\text {A}$ and $F_\text {B}$ using techniques of GSPT generalized for degenerate equilibria (Krupa and Szmolyan 2001; Kuehn 2015). It implies that a trajectory moving rightwards along the slow manifold in chart $K_\text {A}$ (thick black solid line in Fig. 4b) passes the fold point $F_\text {A}$ and transitions to a fast fiber pointing downwards (vertical solid gray line). Similarly, in chart $K_\text {B}$, a trajectory moving leftwards along the slow manifold passes the fold point $F_\text {B}$ and quickly moves vertically upwards, see Fig. 4c. For any fixed ε>0, a coordinate transformation matches the dynamics in charts $K_\text {A}$ and $K_\text {B}$, as discussed below in Sect. 3.3, ultimately resolving the different time scales in the dynamics of the limit cycle. Hence, the local singular perturbation analysis in the two charts $K_\text {A}$ and $K_\text {B}$ is connected into a global analysis for any small ε>0 where, however, the limit $\varepsilon \rightarrow 0$ is not globally resolved due to the singular nature of the rescaling $\eta = h/\varepsilon$. For our purpose, this analysis is sufficient and the transition between $K_\text {A}$ and $K_\text {B}$ does therefore not require additional entry and exit charts, in contrast to a geometric blow-up procedure around one singularity (Krupa and Szmolyan 2001).

### The Fold Point $\boldsymbol{F}_{\textbf{A}}$ Approached from the Acidic Domain, at Low pH

For investigating the turning point ${F}_\text {A}$ located in the acidic domain, at relatively large values of *h* (low pH level), we use the rescaled variables

12 $$\begin{aligned} \sigma = {\varepsilon } s\,, \quad \tau :=\varepsilon t\,, \end{aligned}$$

where σ is a small variable and τ is a slow time relative to the original variables *s* and *t*, respectively. With this ansatz, the dynamical system (9) becomes the fast-slow system

13a $$\begin{aligned} \frac{d\sigma }{d \tau }&= \tilde{f}_\varepsilon (\sigma , h) := - \tilde{r}_\varepsilon (h) \sigma + K_s \,, \end{aligned}$$

13b $$\begin{aligned} \varepsilon \frac{d h}{d \tau }&= \tilde{g}_\varepsilon (\sigma , h) := - \tilde{q}_\varepsilon (\sigma , h) + K_h (1-h) \,, \end{aligned}$$

with $\tilde{r}_\varepsilon (h):= \varepsilon ^{-1} r_\varepsilon (h)$ and $\tilde{q}_\varepsilon (\sigma , h):={q}_\varepsilon (\sigma /\varepsilon , h)$. Inserting the definitions of $r_\varepsilon$ and $q_\varepsilon$ from Eqs. (10a) and (10b), we obtain

14 $$\begin{aligned} \tilde{r}_\varepsilon (h) = \frac{1}{\varepsilon ^2 \beta C/h +\varepsilon + (\beta /C) h} \end{aligned}$$

and

15 $$\begin{aligned} \tilde{q}_\varepsilon (\sigma ,h) = -\frac{1}{2 \varepsilon ^2} \left[ \sqrt{\tilde{v}_\varepsilon (h)^2 + 4 \varepsilon ^2 A K \tilde{r}_\varepsilon (h) h^2 \sigma } - \tilde{v}_\varepsilon (h) \right] , \end{aligned}$$

where

16 $$\begin{aligned} \tilde{v}_\varepsilon (h) := \varepsilon ^2 v_\varepsilon (h)= \alpha A K h^2 -\varepsilon ^2 K_h(1-h) \end{aligned}$$

for $v_\varepsilon (h)$ given in Eq. (11). Switching back to the timescale *t*, we rewrite the fast-slow system (13) as

17a $$\begin{aligned} \frac{d \sigma }{d t }&= \varepsilon \tilde{f}_\varepsilon (\sigma , h)\,, \end{aligned}$$

17b $$\begin{aligned} \frac{d h}{d t}&= \tilde{g}_\varepsilon (\sigma , h)\,. \end{aligned}$$

Here and in the following, the tilde denotes quantities associated with the analysis near the fold point $F_\text {A}$ in the acidic domain.

#### Critical Manifold and Singular Dynamics

The *layer problem* describes the fast, one-dimensional dynamics of h>0 for fixed σ, which acts as a parameter. It is obtained from the fast system Eq. (17) by setting ε=0:

18a $$\begin{aligned} \frac{d \sigma }{d t }&= 0\,, \end{aligned}$$

18b $$\begin{aligned} \frac{d h}{d t }&= \tilde{g}_0(\sigma , h)\,. \end{aligned}$$

However, $\tilde{g}_0(\sigma , h)$ is initially undefined, as $\tilde{q}_\varepsilon (\sigma , h)$ becomes singular at ε=0, see Eq. (15). A continuous extension $\tilde{q}_0(\sigma , h):= \lim _{\varepsilon \rightarrow 0}\tilde{q}_\varepsilon (\sigma , h)$ is obtained by distinguishing the sign of $v_\varepsilon (h)$, which allows us to rewrite $\tilde{q}_\varepsilon (\sigma , h)$ as

19 $$\begin{aligned} \tilde{q}_\varepsilon (\sigma ,h)&= \left\{ \begin{array}{ll} -\dfrac{\tilde{v}_\varepsilon (h)}{2 \varepsilon ^2} \left[ 1 +\sqrt{1 + \varepsilon ^2 4 A K \tilde{r}_\varepsilon (h) h^2 \sigma / \tilde{v}_\varepsilon (h)^2} \right] , & \; \text {if } 0< h < h_\varepsilon ^+ \,, \\ \varepsilon ^{-1}\sqrt{ A K \tilde{r}_\varepsilon (h) h^2 \sigma } , & \; \text {if } h = h_\varepsilon ^+ \,, \\ \dfrac{ 2 A K \tilde{r}_\varepsilon (h) h^2 \sigma }{\tilde{v}_\varepsilon (h)}\left[ 1 + \sqrt{1 + \varepsilon ^2 4 A K \tilde{r}_\varepsilon (h) h^2 \sigma / \tilde{v}_\varepsilon (h)^2} \right] ^{-1}, & \; \text {if } h > h_\varepsilon ^+ \,, \end{array} \right. \end{aligned}$$

in terms of the positive root $h_\varepsilon ^+$ of the equation $v_\varepsilon (h)=0$:

20 $$\begin{aligned} h_\varepsilon ^+ = \varepsilon \, \frac{-\varepsilon K_h+\sqrt{ (\varepsilon K_h)^2 + 4 \alpha A K K_h}}{2 \alpha A K} \,. \end{aligned}$$

Thus, $h_\varepsilon ^+\rightarrow 0$ as $\varepsilon \rightarrow 0$, and for all h>0 the following holds:

21 $$\begin{aligned} \tilde{q}_0(\sigma ,h) = \frac{A K \tilde{r}_0(h) h^2 \sigma }{\tilde{v}_0(h)} = \frac{C \sigma }{\alpha \beta h}, \end{aligned}$$

upon inserting $\tilde{r}_0(h)= C/(\beta h)$ and $\tilde{v}_0(h)=\alpha A K h^2$, see Eqs. (14) and (16).

The so-called *reduced problem* is obtained from Eq. (13) by setting ε=0; it has the form of a differential-algebraic equation:

22a $$\begin{aligned} \frac{d \sigma }{d \tau }&= \tilde{f}_0(\sigma , h) := -\frac{C \sigma }{\beta h} +K_s \,, \end{aligned}$$

22b $$\begin{aligned} 0&= \tilde{g}_0(\sigma , h) := - \frac{C \sigma }{\alpha \beta h} + K_h(1-h) \,. \end{aligned}$$

The *critical manifold* consists of the equilibria of the layer problem, Eq. (18b), and is determined by the zeros of $\tilde{g}_0$, given by

23 $$\begin{aligned} \tilde{\mathcal {S}}_\text {A} = \bigl \{ (\varphi (h), h): \, h > 0 \bigr \} \quad \text {with} \quad \varphi (h) := \frac{\alpha \beta K_h}{C}\, h (1-h) \,. \end{aligned}$$

The function φ(h) possesses only one extremum at h=1/2, which corresponds to the acidic fold point

24 $$\begin{aligned} F_\text {A} := (\sigma _\text {A}, h_\text {A}) = (\varphi (h_\text {A}),h_\text {A}) = \left( \frac{\alpha \beta K_h }{4 C}, \frac{1}{2} \right) \,. \end{aligned}$$

**Fig. 5:**
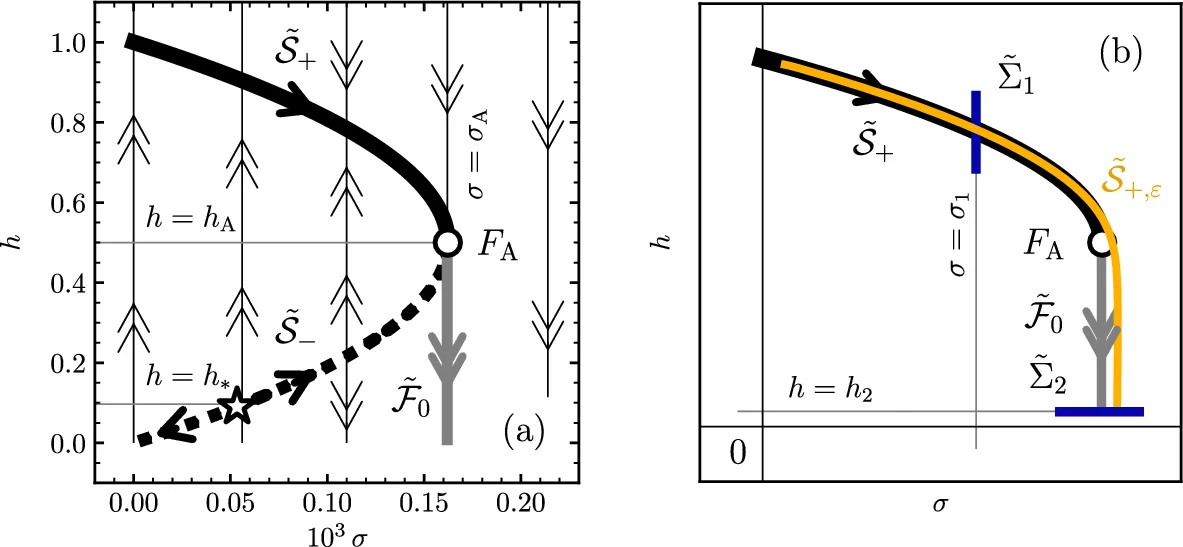
**a** The critical manifold $\tilde{\mathcal {S}}_\text {A}=\tilde{\mathcal {S}}_+ \cup \{ F_\text {A} \} \cup \tilde{\mathcal {S}}_-$ (thick black lines) consists of the fold point $F_\text {A}$ (open circle, Eq. (24)), the attractive branch $\tilde{\mathcal {S}}_+$ (solid line) and the repelling branch $\tilde{\mathcal {S}}_-$ (dotted line), see Eq. (23). The slow motion along $\tilde{\mathcal {S}}_+$ is governed by Eq. (27) and progresses towards the generic fold point $F_\text {A}$, where a loss of hyperbolicity triggers a transition to fast motion along the fast fiber. The segment of the fast fiber extending downward from $F_\text {A}$, denoted as $\tilde{\mathcal {F}}_0$ (thick gray line), is given by Eq. (18b) for $\sigma =\sigma _\text {A}$. The thin horizontal gray lines indicate $h=h_\text {A}$ and $h=h_*$; the star indicates the fixed point of system (22). **b** Schematic of the ε-perturbed extension (solid yellow line) of the critical manifold from panel **a**, following $\tilde{\mathcal {S}}_{+,\varepsilon }$ and passing near the fold point $F_\text {A}$. The Poincaré sections $\tilde{\Sigma }_1$ and $\tilde{\Sigma }_2$ are shown as dark blue lines. The thin gray lines are for $\sigma =\sigma _1$ and $h=h_2$, respectively; the remaining symbols are as in **a** (Color figure online)

The stability of the manifold $\tilde{\mathcal {S}}_\text {A}$ is determined by the sign of the derivative $\partial _{h} \tilde{g}_0(\sigma , h)$ on $\tilde{\mathcal {S}}_A$:

25 $$\begin{aligned} \partial _{h} \tilde{g}_0(\sigma , h)\Big |_{(\sigma , h) \in \tilde{\mathcal {S}}_\text {A}}&= \partial _{h} \left( - \frac{C \sigma }{\alpha \beta h} + K_h(1-h)\right) \Bigg |_{(\sigma , h) \in \tilde{\mathcal {S}}_\text {A}} \nonumber \\&= - 2 K_h \frac{h - h_\text {A}}{h} \,. \end{aligned}$$

In particular, $\partial _{h} \tilde{g}_0$ changes sign at $F_\text {A}$. The critical manifold $\tilde{\mathcal {S}}_\text {A}$ contains the sets^1^$\tilde{\mathcal {S}}_+$ and $\tilde{\mathcal {S}}_-$, where $\tilde{\mathcal {S}}_+$ represents the attractive upper branch for $h > h_\text {A}$, characterized by $\partial _{h} \tilde{g}_0(\sigma , h) < 0$, and $\tilde{\mathcal {S}}_-$ represents the repelling lower branch for $h < h_\text {A}$, characterized by $\partial _{h} \tilde{g}_0(\sigma , h) > 0$, see Fig. 5a. The manifold is therefore normally hyperbolic everywhere except at the fold point $F_\text {A}$.

The slow dynamics along the critical manifold $\tilde{\mathcal {S}}_\text {A}$ is given by the reduced problem, Eq. (22). Substituting φ(h) via Eq. (23) in Eq. (22a) yields:

26 $$\begin{aligned} \frac{d \sigma }{d \tau }\bigg |_{(\sigma ,h) \in \tilde{\mathcal {S}}_\text {A}} = \left( K_s-\frac{C\sigma }{\beta h}\right) \bigg |_{\sigma =\varphi (h)} = \alpha K_h(h-h_*) \,, \end{aligned}$$

where $h_*$ is given by Eq. (6); in Fig. 5a, the fixed point $(K_s/\tilde{r}_0(h_*), h_*)$ of Eq. (22) is shown as a star. Using $d \sigma /d \tau =d \varphi (h)/d \tau =\varphi '(h) \, d h/d \tau$ and the derivative $\varphi '(h)=(\alpha K_h \beta /C) (1-h/h_\text {A})$, yields

27 $$\begin{aligned} \left. \frac{d h}{ d \tau }\right| _{(\sigma ,h) \in \tilde{\mathcal {S}}_\text {A}}&= \left. \frac{d \sigma }{ d \tau } \, \frac{1}{\varphi '(h)} \right| _{(\sigma ,h) \in \tilde{\mathcal {S}}_\text {A}} = \left( h - 1 + \frac{K_s}{\alpha K_h} \right) \, \frac{C/\beta }{1-h/h_\text {A}} \nonumber \\&= -\frac{C h_\text {A}(h-h_*)}{\beta (h - h_\text {A})} \,, \end{aligned}$$

Both, $d\sigma /d\tau$ and dh/dτ flip sign at $h=h_*$. Restricting to $h > h_*$ and since $h_\text {A} > h_*$, the sign of the right-hand side of Eq. (27) is determined by the difference $h-h_\text {A}$ and changes at $F_\text {A}$. For $h > h_\text {A}$, the motion along the upper branch of the critical manifold occurs with dh/dτ<0 from smaller to larger values of σ, whereas dh/dτ>0 for motion on the lower branch, $h_*< h < h_\text {A}$, also see Fig. 5a. These observations indicate that, in both cases, the motion along the attractive branch $\tilde{\mathcal {S}}_+$ and the repelling branch $\tilde{\mathcal {S}}_-$ for any $\sigma < \sigma _\text {A}$ is always towards the non-hyperbolic point $F_\text {A}$ (in its vicinity).

##### Proposition 2

The critical manifold $\tilde{\mathcal {S}}_\text {A}$ possesses a single fold point, given by $F_\text {A}$ in Eq. (24), and this point is a generic fold; in particular, it is nondegenerate and the flow at $F_\text {A}$ is transversal to $\tilde{\mathcal {S}}_\text {A}$ (see Appendix B).

##### Proof

We have already observed that

28 $$\begin{aligned} \tilde{g}_0(F_\text {A}) = 0 \quad \text {and} \quad \partial _{h}\tilde{g}_0(F_\text {A})=0, \end{aligned}$$

indicating that $F_\text {A}$ is a fold point; it is also the only such point on $\tilde{\mathcal {S}}_\text {A}$, see Eq. (22b).

It remains to verify the nondegeneracy and transversality conditions on $F_\text {A}$. Indeed, we find from Eqs. (22) and (25) that

29 $$\begin{aligned} \partial _{h}^2 \tilde{g}_0(F_\text {A}) = - 4 K_h< 0, \qquad \partial _{\sigma } \tilde{g}_0(F_\text {A}) = - \frac{2 C}{\alpha \beta } < 0, \end{aligned}$$

and

30 $$\begin{aligned} \tilde{f}_0(F_\text {A})=\alpha K_h(h_\text {A}-h_*)>0, \end{aligned}$$

using $h_* > 0$ from Eq. (6). The conditions in Eq. (29) establish the nondegeneracy of the fold point. Together with the transversality condition, Eq. (30), this confirms that $F_\text {A}$ is a generic fold point. □

#### Extension of Slow Manifolds Through the Fold Point

Recall that the stable branch $\tilde{\mathcal {S}}_+$ of the critical manifold is normally hyperbolic for all $h > h_\text {A}$. By the smoothness of the system, Fenichel’s theorem guarantees that for any small $\gamma ,\delta >0$, the compact segment

$$\begin{aligned} \{ (\varphi (h), h): h \in [h_\text {A} + \gamma , 1- \delta ] \} \subset \tilde{\mathcal {S}}_+ \end{aligned}$$

gives rise to a nearby *slow manifold* $\tilde{\mathcal {S}}_{+,\varepsilon }$ for sufficiently small ε>0, lying within an $\mathcal {O}(\varepsilon )$-neighborhood of the critical manifold. Although uniqueness is not guaranteed, any such slow manifold can be written, up to first-order approximation, as the graph of a function $\varphi _\varepsilon (h)= \varphi (h)+ \mathcal {O}(\varepsilon )$.

Standard GSPT ensures that any slow manifold can be extended beyond the fold point $F_\text {A}$, provided it is nondegenerate. Such an extension is illustrated in Fig. 5b for the dynamical system (17). Specifically, we consider the Poincaré sections

31 $$\begin{aligned} \tilde{\Sigma }_1:= \{ (\sigma , h) : \sigma =\sigma _1,\; h\in \tilde{I} \}, \qquad \tilde{\Sigma }_2:= \{ (\sigma , h) : |\sigma -\sigma _\text {A}|\leqslant \delta ,\; h=h_2\} \end{aligned}$$

for some $0< \sigma _1 < \sigma _\text {A}$, $0< h_2 < h_\text {A}$, and an appropriate compact interval $\tilde{I}$, such that $\tilde{\mathcal {S}}_+ \cap \tilde{\Sigma }_1$ is not empty. The extension of $\tilde{\mathcal {S}}_{+,\varepsilon }$ beyond the fold point follows from the next proposition, which implies that, for any ε>0 sufficiently small, also the intersection $\tilde{\mathcal {S}}_{+,\varepsilon } \cap \tilde{\Sigma }_1$ is nonempty and given by a point $(\sigma _1,\varphi (\sigma _1)) + \mathcal {O}(\varepsilon )$.

*Transition map.* Given two transversal sections $\tilde{\Sigma }_1$ and $\tilde{\Sigma }_2$, the *transition map* $\Pi ^{(\varepsilon )}_{1,2}: \tilde{\Sigma }_1 \rightarrow \tilde{\Sigma }_2$ assigns to each point $(\sigma , h) \in \tilde{\Sigma }_1$ the first intersection point of the corresponding trajectory of the system (depending on ε) with $\tilde{\Sigma }_2$. That is, $\Pi ^{(\varepsilon )}_{1,2}(\sigma , h)$ gives the location where the trajectory starting at (σ,h) reaches $\tilde{\Sigma }_2$. This map captures the evolution of trajectories between two phase space sections, which is central to understanding the global behavior of fast–slow systems.

Since $F_\text {A}$ is a generic fold point (Prop. 2), the following statement is a direct consequence of Theorem 2.1 by Krupa and Szmolyan Krupa and Szmolyan (2001). In particular, the transversality condition, Eq. (30), precludes the existence of a canard trajectory passing through $F_\text {A}$.

##### Proposition 3

For appropriate sections $\tilde{\Sigma }_1, \tilde{\Sigma }_2$ as defined above, the transition map $\Pi _{1,2}^{(\varepsilon )}:\tilde{\Sigma }_1\rightarrow \tilde{\Sigma }_2$ is well-defined for sufficiently small ε>0, and acts as an $\mathcal {O}(e^{-c_1/\varepsilon })$-contraction for some $c_1>0$. Furthermore, for any $P\in \tilde{\Sigma }_1$, the image under the transition map satisfies

32 $$\begin{aligned} \Pi _{1,2}^{(\varepsilon )}(P) = \left( \sigma _\text {A}+\mathcal {O}\left( \varepsilon ^{2/3}\right) ,h_2\right) . \end{aligned}$$

### The Fold Point $\boldsymbol{F}_{\textbf{B}}$ Approached from the Basic Domain, at High pH

The candidate for a second fold point, later identified as $F_\text {B}$, corresponds to nearly neutral $\hbox {pH}$ and is approached from the basic domain at $h \simeq \varepsilon C$ as $\varepsilon \rightarrow 0$ (cf. Eq. (45) below) and, in the limit, is pushed against the h=0 axis (high pH level), hindering its analysis. To better resolve the properties of this point, we transform the dynamical system (9) by rescaling the *h* variable as

33 $$\begin{aligned} \eta :=h/\varepsilon \,, \end{aligned}$$

which effectively zooms into the vicinity of the h=0 line. As a result, we arrive at the *fast-slow system*:

34a $$\begin{aligned} \frac{ds}{dt}&= \hat{f}_\varepsilon (s, \eta ) := -\hat{r}(\eta ) s + K_s \,, \end{aligned}$$

34b $$\begin{aligned} \varepsilon \frac{d \eta }{dt}&= \hat{g}_\varepsilon (s,\eta ) := -\hat{q}_\varepsilon (s, \eta ) + K_h (1-\varepsilon \eta ) \,, \end{aligned}$$

with the rescaled functions $$\hat{r}(\eta ):= r_\varepsilon (\varepsilon \eta )$$ and $$\hat{q}_\varepsilon (s, \eta ):= q_\varepsilon (s,\varepsilon \eta )$$, see Eq. (10). Introducing the fast time $t^{\prime }=t/\varepsilon$, the fast-slow system (34) takes the form:

35a $$\begin{aligned} \frac{ds}{d t'}&= \varepsilon \hat{f}_\varepsilon (s,\eta )\,, \end{aligned}$$

35b $$\begin{aligned} \frac{d \eta }{d t'}&= \hat{g}_\varepsilon (s,\eta ) \,. \end{aligned}$$

In analogy to the tilde notation used near $F_A$, the hat denotes quantities associated with the analysis near the fold point $F_B$. For later use, we list the functions appearing on the right hand side of Eq. (34) for ε=0:

36 $$\begin{aligned} \hat{r}(\eta ) = \frac{1}{\beta C \eta ^{-1} + 1 + (\beta /C) \eta } \,, \end{aligned}$$

which does not depend on ε, and

37 $$\begin{aligned} \hat{q}_0(s,\eta ) = \frac{1}{2}\sqrt{\hat{v}_0(\eta )^2 + 4 A K \hat{r}(\eta ) \eta ^2 s } - \frac{\hat{v}_0(\eta )}{2} \,, \end{aligned}$$

with

38 $$\begin{aligned} \hat{v}_0(\eta ) := v_\varepsilon (\varepsilon \eta )|_{\varepsilon =0} = \alpha A K \eta ^2 -K_h \,. \end{aligned}$$

#### Critical Manifold and Singular Dynamics

The corresponding *layer problem* is a one-dimensional dynamics in the fast variable η, parametrized by constant values of *s*. It is obtained by setting ε=0 in (35):

39a $$\begin{aligned} \frac{ds}{d t'}&= 0\,, \end{aligned}$$

39b $$\begin{aligned} \frac{d \eta }{d t'}&=\hat{g}_0(s,\eta )\, . \end{aligned}$$

The *reduced problem* follows by setting ε=0 in (34) and reads:

40a $$\begin{aligned} \frac{ds}{dt}&= \hat{f}_0(s,\eta ) := -\hat{r}(\eta ) s + K_s \,, \end{aligned}$$

40b $$\begin{aligned} 0&= \hat{g}_0(s,\eta ) := -\hat{q}_0(s,\eta ) + K_h \,. \end{aligned}$$

The *critical manifold* is determined by the algebraic equation (40b):

41 $$\begin{aligned} \hat{\mathcal {M}}_\text {B}&:= \{ (s, \eta ): \, \hat{g}_0(s, \eta ) = 0 \} \nonumber \\&= \left\{ (s, \eta ): (\alpha K_h - \hat{r}(\eta )s)\eta ^2 = 0 \right\} \,; \end{aligned}$$

the second line follows from the equation $2 \hat{q}_0(s,\eta ) + \hat{v}_0(\eta ) = 2 K_h + \hat{v}_0(\eta )$ after substituting Eqs. (37) and (38) and taking the square. In particular, it is the union $\hat{\mathcal {M}}_\text {B} = \hat{\mathcal {S}}_\text {B} \cup \hat{\mathcal {L}}_\text {B}$ of a curve $\hat{\mathcal {S}}_\text {B}$ in the domain η>0,

42 $$\begin{aligned} \hat{\mathcal {S}}_\text {B} = \bigl \{ (\psi (\eta ), \eta ) : \eta > 0 \bigr \} \quad \text {with} \quad \psi (\eta ) := \frac{\alpha K_h}{\hat{r}(\eta )} \,, \end{aligned}$$

and the *s*-axis, where η=0:

43 $$\begin{aligned} \hat{\mathcal {L}}_\text {B} = \bigl \{ (s, 0) : s \geqslant 0 \bigr \}\,. \end{aligned}$$

Inspection of the derivative

44 $$\begin{aligned} \psi '(\eta ) = \alpha K_h (\beta /C) (1-C^2/\eta ^2) \end{aligned}$$

shows that the branch $\hat{\mathcal {S}}_\text {B}$ has a unique fold point,

45 $$\begin{aligned} F_\text {B}:= (s_\text {B}, \eta _\text {B}) = (\psi (\eta _\text {B}),\eta _\text {B}) = \left( \frac{\alpha K_h}{ \hat{r}(\eta _\text {B})}, C \right) , \end{aligned}$$

where one can further evaluate $\hat{r}(\eta _\text {B})=1/(2\beta +1)$.

The stability of the critical manifold is inferred from the derivative of $\hat{g}_0$ along $\hat{\mathcal {S}}_\text {B}$. Using that $\hat{g}_0$ is constant on $\hat{\mathcal {S}}_\text {B}$, it holds

46 $$\begin{aligned} 0 = d \hat{g}_0(s,\eta ) = \partial _s \hat{g}_0(s,\eta ) \, ds + \partial _\eta \hat{g}_0(s,\eta ) \, d\eta \,; \quad (s,\eta ) \in \hat{\mathcal {S}}_\text {B}. \end{aligned}$$

Observing further that

47 $$\begin{aligned} \partial _s \hat{g}_0(s,\eta ) = - \frac{A K \hat{r}(\eta ) \eta ^2}{2 \hat{q}_0(s,\eta ) + \hat{v}_0(\eta )} \,, \end{aligned}$$

one readily computes

48 $$\begin{aligned} \partial _{\eta } \hat{g}_0(s, \eta )\big |_{(s, \eta ) \in \hat{\mathcal {S}}_\text {B}}&= -\partial _{s} \hat{g}_0(s, \eta ) \, \psi '(\eta ) \big |_{(s, \eta ) \in \hat{\mathcal {S}}_\text {B}} \nonumber \\&= \frac{(\beta /C) K_h \hat{r}(\eta )}{\eta ^2 + K_h/(\alpha A K)}\left( \eta ^2 - \eta _\text {B}^2 \right) , \end{aligned}$$

after inserting Eq. (44) and $\eta _\text {B} = C$.

**Fig. 6:**
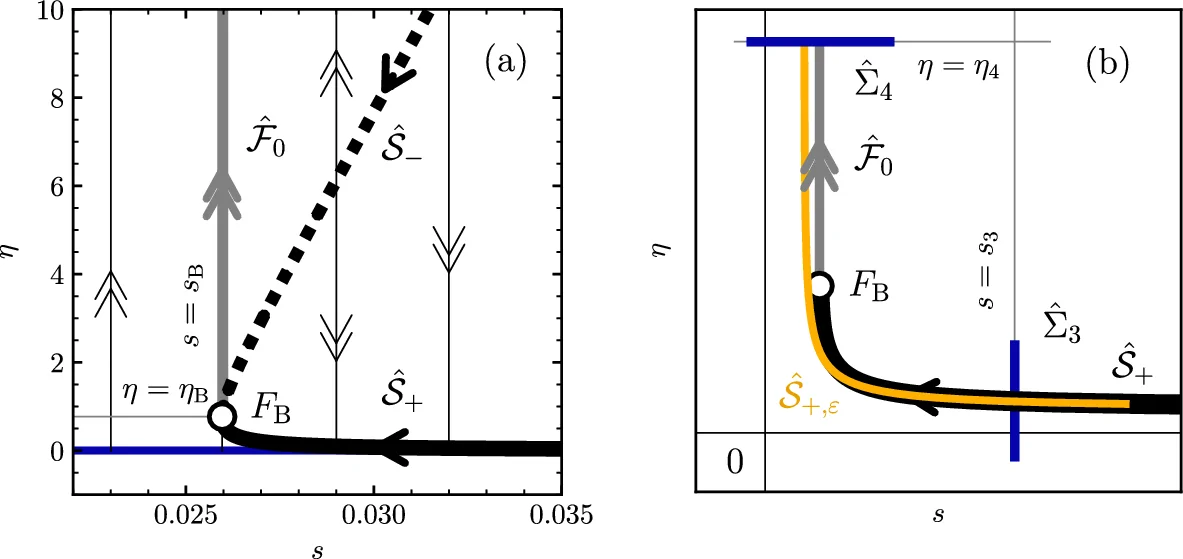
**a** The nontrivial part $\hat{\mathcal {S}}_\text {B}=\hat{\mathcal {S}}_+ \cup \{ F_\text {B} \} \cup \hat{\mathcal {S}}_-$ (thick black lines) of the critical manifold $\hat{\mathcal {M}}_{\text {B}}$ consists of the fold point $F_\text {B}$ (circle, Eq. (45)), the attractive branch $\hat{\mathcal {S}}_+$ (solid line), and the repelling branch $\hat{\mathcal {S}}_-$ (dotted line), see Eq. (42). The trivial part $\hat{\mathcal {L}}_\text {B}$ of $\hat{\mathcal {M}}_{\text {B}}$ is the axis η=0 (blue line). The slow motion along $\hat{\mathcal {S}}_+$ is governed by Eq. (50) and progresses left towards the generic fold point $F_\text {B}$, where a loss of hyperbolicity induces the switching to fast motion along the fast fiber. The segment of the fast fiber extending upwards from $F_\text {B}$, denoted as $\hat{\mathcal {F}}_0$ (thick gray line) is described by Eq. (39b) with $s=s_\text {B}$. The horizontal, thin gray line marks $\eta =\eta _\text {B}$. **b** Schematic of the ε-perturbed extension (solid yellow line) of the critical manifold from panel **a**, following $\hat{\mathcal {S}}_{+,\varepsilon }$ and passing near the fold point $F_\text {B}$. The Poincaré sections $\hat{\Sigma }_3$ and $\hat{\Sigma }_4$ are shown as dark blue lines; the thin gray lines are for $s=s_3$ and $\eta =\eta _4$, respectively; the remaining symbols are as in **a**(Color figure online)

Hence, the stability of the manifold $\hat{\mathcal {S}}_\text {B}$ depends on the sign of $\eta -\eta _\text {B}$: $\hat{\mathcal {S}}_\text {B}$ is stable for $\eta < \eta _\text {B}$ and unstable for $\eta > \eta _\text {B}$. Stated differently: $\hat{\mathcal {S}}_\text {B}$ is normally hyperbolic everywhere, except for the point $F_\text {B}$, where hyperbolicity is lost. Similarly to the manifold $\tilde{\mathcal {S}}_\text {A}$, with its fold point $F_\text {A}$, the manifold $\hat{\mathcal {S}}_\text {B}$ is divided at $F_\text {B}$ into an attractive branch $\hat{\mathcal {S}}_+$, below $F_\text {B}$, and a repelling branch^2^$\hat{\mathcal {S}}_-$, above $F_\text {B}$, which meet at the fold point, see Fig. 6a.

The reduced flow on the critical manifold $\hat{\mathcal {S}}_\text {B}$ follows from Eq. (42) with Eq. (40a):

49 $$\begin{aligned} \left. \frac{ds}{dt}\right| _{(s, \eta ) \in \hat{\mathcal {S}}_B} = (-\hat{r}(\eta )s + K_s)|_{s = \psi (\eta )} = -\alpha K_h h_* < 0 \,,\end{aligned}$$

recalling $h_*$ from Eq. (6). For the fast variable, we use $ds/dt = d\psi (\eta )/dt = \psi '(\eta ) \, d\eta /dt$ and Eq. (44) to find:

50 $$\begin{aligned} \frac{d \eta }{ d t}\bigg |_{(s, \eta ) \in \hat{\mathcal {S}}_\text {B}}&= \frac{d s}{d t} \, \frac{1}{\psi '(\eta )} \bigg |_{(s,\eta ) \in \hat{\mathcal {S}}_\text {B}} = \frac{C h_*}{\beta } \frac{\eta ^2}{\eta _\text {B}^2-\eta ^2} \,. \end{aligned}$$

Since C/β>0 and $h_* > 0$, the sign of dη/dt on $\hat{\mathcal {S}}_\text {B}$ is equal to the sign of $\eta _\text {B}-\eta$. Starting a trajectory on the manifold at $\eta < \eta _\text {B}$, it evolves towards larger values of η and is accelerated upon approaching the singular point $F_\text {B}$, whereas the value of η decreases upon starting at $\eta > \eta _\text {B}$; in both cases, the value of *s* decreases with constant speed. In other words, the motion along the attractive branch $\hat{\mathcal {S}}_+$ and the repelling branch $\hat{\mathcal {S}}_-$ for any $s > s_\text {B}$ is always towards $F_\text {B}$, see Fig. 6a. These observations are consistent with the following statement:

##### Proposition 4

The critical manifold $\hat{\mathcal {S}}_\text {B}$ possesses a single fold point, given by $F_\text {B}$ in Eq. (45), and this point is a generic fold.

##### Proof

We have already seen from Eq. (48) that $F_\text {B} \in \hat{\mathcal {S}}_\text {B}$ is a fold point, uniquely satisfying $\hat{g}_0(F_\text {B}) = 0$ and $\partial _{\eta }\hat{g}_0(F_\text {B})=0$. For the nondegeneracy of $F_\text {B}$, one verifies the stability along the fast direction,

51 $$\begin{aligned} \partial _\eta ^2 \hat{g}_0(F_\text {B})&= \frac{ (\beta /C) K_h \hat{r}(\eta _\text {B})}{\eta _\text {B}^2 +K_h/(\alpha A K)} \, \partial _\eta \left( \eta ^2 -\eta _\text {B}^2\right) \Big |_{\eta =\eta _B} \nonumber \\&= 2\eta _\text {B} \frac{(\beta /C) K_h \hat{r}(\eta _\text {B})}{\eta _\text {B}^2 +K_h/(\alpha A K)}>0\,, \end{aligned}$$

and regularity along the slow direction,

52 $$\begin{aligned} \partial _s \hat{g}_0(F_\text {B})&= -\frac{ \hat{r}(\eta _\text {B})}{ \alpha + K_h/(A K \eta _\text {B}^2) } < 0 \,. \end{aligned}$$

The first equation follows from Eq. (48), and we have used Eq. (47) with Eq. (38) to obtain the second relation. Finally, the transversality condition can be read off from Eq. (49):

53 $$\begin{aligned} \hat{f}_0(F_\text {B})=- \alpha K_h h_* < 0 \,. \end{aligned}$$

Hence, $F_\text {B}$ is a generic fold point. □

#### Extension of Slow Manifolds Through the Fold Point

Similarly to the situation of $\tilde{\mathcal {S}}_\text {A}$, any compact submanifold of $\hat{\mathcal {S}}_+$ persists for ε sufficiently small, and can be extended beyond $F_\text {B}$. Indeed, let

54 $$\begin{aligned} \hat{\Sigma }_3 := \{ (s,\eta ) : s = s_3, \;\eta \in \hat{I} \}, \qquad \hat{\Sigma }_4:=\left\{ (s, \eta ): \, |s-s_\text {B}| \leqslant \delta , \eta = \eta _4 \right\} \end{aligned}$$

for some $s_3>s_\text {B}$, $\eta _4>\eta _\text {B}$ and an appropriate compact interval $\hat{I}$; see also Fig. 6b. Analogously to Sect. 3.1.2, the extension of any slow manifold $\hat{\mathcal {S}}_{+,\varepsilon }$ beyond the generic fold point $F_\text {B}$ (Prop. 4) is given by the following statement, which is granted by Theorem 2.1 of Krupa and Szmolyan (2001). Again, a nonvanishing value of the flow in Eq. (53) excludes the possibility of canards at the point $F_\text {B}$.

##### Proposition 5

For appropriate sections $\hat{\Sigma }_3$ and $\hat{\Sigma }_4$ as defined in Eq. (54), the transition map $\Pi _{3,4}^{(\varepsilon )}:\hat{\Sigma }_{3}\rightarrow \hat{\Sigma }_{4}$ is well-defined for sufficiently small ε>0, and it acts as an $\mathcal {O}(e^{-c_2/\varepsilon })$-contraction for some $c_2>0$. Furthermore, for any $P\in \hat{\Sigma }_3$, the image under the transition map satisfies

55 $$\begin{aligned} \Pi _{3,4}^{(\varepsilon )}(P) = (s_\text {B},\eta _4) + \mathcal {O}\left( \varepsilon ^{2/3}\right) . \end{aligned}$$

### Global Picture Underlying the Formation of the Limit Cycle

So far, we have clarified how two different scalings—essentially represented in Fig. 4 by the charts $K_\text {A}$ and $K_\text {B}$, operating in different coordinates (σ,h) and (s,η), respectively—resolve the dynamics around the fold points $F_\text {A}$ and $F_\text {B}$. The existence of an attracting limit cycle was already established in Sect. 2.2, and it now remains to connect these two perspectives to a global picture, revealing the formation and overall structure of the limit cycle. Our findings from the fast–slow analysis are then illustrated and corroborated by numerical solutions for limit cycle trajectories along the ε-perturbed slow manifolds and fast fibers.

#### Constructing the Limit Cycle from Transition Maps

**Fig. 7:**
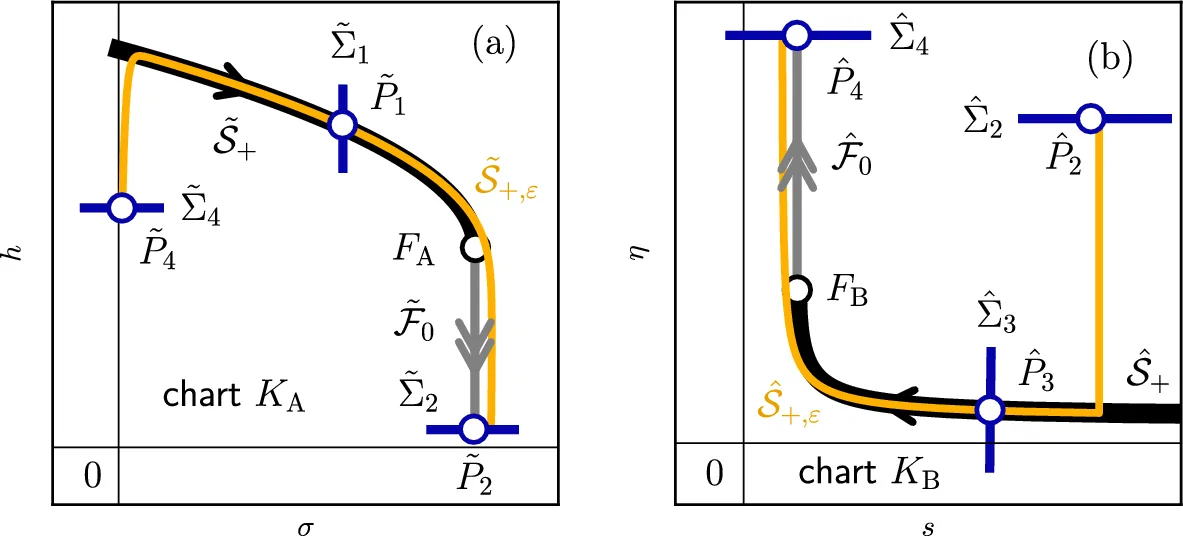
Composition of the limit cycle from transition maps within and between the charts $K_\text {A}$ and $K_\text {B}$, as described in Sect. 3.3. The two panels depict the slow attractive manifolds $\tilde{\mathcal {S}}_+$, $\hat{\mathcal {S}}_+$ (solid black line), the fast fibers $\tilde{\mathcal {F}}_0$, $\hat{\mathcal {F}}_0$ (solid gray lines) in the singular limit, an ε-perturbed trajectory (solid yellow lines), and suitable Poincaré sections (dark blue lines). **a** Chart $K_\text {A}$ uses coordinates (σ,h) to show the manifold $\tilde{\mathcal {S}}_+ \cup \tilde{\mathcal {F}}_0$, which determines the passage through the fold point $F_\text {A}$, and Poincaré sections $\tilde{\Sigma }_1$, $\tilde{\Sigma }_2$, $\tilde{\Sigma }_4$; with their intersection points $\tilde{P}_1 = \tilde{\Sigma }_1 \cap \tilde{\mathcal {S}}_+$ and $\tilde{P}_2 = \tilde{\Sigma }_2 \cap \tilde{\mathcal {F}}_0$. The point $\tilde{P}_4$ is identified with $\hat{P}_4$ in chart $K_\text {B}$. **b** Chart $K_\text {B}$ uses coordinates (s,η) to show the manifold $\hat{\mathcal {S}}_+ \cup \hat{\mathcal {F}}_0$, which determines the passage through the fold point $F_\text {B}$, and Poincaré sections $\hat{\Sigma }_2$, $\hat{\Sigma }_3$, $\hat{\Sigma }_4$ with intersection points $\hat{P}_3 = \hat{\Sigma }_3 \cap \hat{\mathcal {S}}_+$ and $\hat{P}_4 = \hat{\Sigma }_4 \cap \hat{\mathcal {F}}_0$. The point $\hat{P}_2$ is identified with $\tilde{P}_2$ in chart $K_\text {A}$ (Color figure online)

Building on the results of Sects. 3.1 and 3.2, we construct a sequence of Poincaré sections and their corresponding transition maps, as illustrated in Fig. 7:

$$\begin{aligned} \Pi _{1,2}^{(\varepsilon )}&: \tilde{\Sigma }_1 \rightarrow \tilde{\Sigma }_2 &  \text {for the passage of the fold point} F_\text {A}=(\sigma _\text {A}, h_\text {A}) \text {in chart} K_\text {A}, \\ \Phi _{\text {A}, \text {B}}&: \tilde{\Sigma }_2 \rightarrow \hat{\Sigma }_2 &  \text {for the transformation from chart} K_\text {A} \text {to} K_\text {B}, \\ \Pi _{2,3}^{(\varepsilon )}&: \hat{\Sigma }_2 \rightarrow \hat{\Sigma }_3 &  \text {for the transition towards the slow attracting manifold} \hat{\mathcal {S}}_+ \text {in chart} K_\text {B}, \\ \Pi _{3,4}^{(\varepsilon )}&: \hat{\Sigma }_3 \rightarrow \hat{\Sigma }_4 &  \text {for the passage of the fold point} F_\text {B}=(s_\text {B}, \eta _\text {B}) \text {in chart } K_\text {B}, \\ \Phi _{\text {B}, \text {A}}&: \hat{\Sigma }_4 \rightarrow \tilde{\Sigma }_4 &  \text {for the transformation from chart } K_\text {B} \text { to } K_\text {A}, \\ \Pi _{4,1}^{(\varepsilon )}&: \tilde{\Sigma }_4 \rightarrow \tilde{\Sigma }_1 &  \text {for the transition towards the slow attracting manifold } \tilde{\mathcal {S}}_+ \text {in chart} K_\text {A}. \end{aligned}$$

Here, $\hat{\Sigma }_2$ is the same as $\tilde{\Sigma }_2$ after a coordinate transformation from (σ,h) to (s,η), and vice versa; $\tilde{\Sigma }_4$ is $\hat{\Sigma }_4$ in (σ,h)-coordinates. The individual steps are described in detail below. One full loop of the limit cycle is captured by the composition $\Pi : \tilde{\Sigma }_1 \rightarrow \tilde{\Sigma }_1$ of the above transition maps:

56 $$\begin{aligned} \Pi = \Pi _{1,2}^{(\varepsilon )} \circ \Phi _{\text {A}, \text {B}} \circ \Pi _{2,3}^{(\varepsilon )} \circ \Pi _{3,4}^{(\varepsilon )} \circ \Phi _{\text {B}, \text {A}} \circ \Pi _{4,1}^{(\varepsilon )}\,. \end{aligned}$$

We note that working with an arbitrarily small, but nonzero ε>0 is crucial for ensuring well-defined transformations between the charts $K_\text {A}$ and $K_\text {B}$.

#### Description of $\Pi _{1,2}^{(\varepsilon )}$

The transition $\Pi _{1,2}^{(\varepsilon )}$ occurs within chart $K_\text {A}$ and was characterized in Prop. 3. As detailed in Sect. 3.1.1, the upper branch $\tilde{\mathcal {S}}_+$ of the slow critical manifold $\mathcal {S}_\text {A}$ is quickly attracting. In the singular limit, ε=0, the intersection of $\tilde{\Sigma }_1$ with $\tilde{\mathcal {S}}_+$, see Fig. 5b, is well-defined and corresponds to a certain point $\tilde{P}_1:=(\sigma _1, h_1)=(\varphi (h_1), h_1)$ for some $h_1>0$, with φ defined in Eq. (23). From $\tilde{P}_1$, the trajectory follows the slow manifold $\tilde{\mathcal {S}}_+$ up to the fold point $F_\text {A}=(\sigma _\text {A},h_\text {A})$ (cf. Eq.  (24)), as described in Sect. 3.1.1, and then transitions to and along the fast fiber $\tilde{\mathcal {F}}_0$, Eq. (18b) with $\sigma =\sigma _\text {A}$. As a result, the trajectory follows the composite manifold $\tilde{\Gamma }_0:= \tilde{\mathcal {S}}_+ \cup \tilde{\mathcal {F}}_0$ and arrives at the point $\tilde{P}_2:= (\sigma _\text {A}, h_2) \in \tilde{\Sigma }_2$ for the Poincaré section $\tilde{\Sigma }_2$ defined in Eq. (31).

For the ε-perturbed case, the trajectory begins at a slightly shifted point, $\tilde{P}_{1,\varepsilon } \in \tilde{\Sigma }_1 \cap \tilde{\mathcal {S}}_{+,\varepsilon }$, follows the perturbed manifold $\tilde{\Gamma }_\varepsilon = \tilde{\mathcal {S}}_{+,\varepsilon } \cup \tilde{\mathcal {F}}_\varepsilon$, and reaches $\tilde{P}_{2,\varepsilon }:= (\sigma _{\text {A},\varepsilon }, h_2) \in \tilde{\Sigma }_2$. Both the fast fiber $\tilde{\mathcal {F}}_0$ and its perturbed continuation $\tilde{\mathcal {F}}_\varepsilon$ are transverse to $\tilde{\Sigma }_2$, ensuring that the transition map $\Pi _{1,2}^{(\varepsilon )}$ is well-defined.

#### Description of $\Phi _{\text {A}, \text {B}}$

The transformation $\Phi _{\text {A}, \text {B}}: \tilde{\Sigma }_2 \rightarrow \hat{\Sigma }_2$ facilitates the switch from the chart $K_\text {A}$ with the coordinates (σ,h) to chart $K_\text {B}$ with the coordinates (s,η) such that $\sigma = \varepsilon s$ and $\eta =h/\varepsilon$, see Eqs. (12) and (33). Hence, the Poincaré section $\tilde{\Sigma }_2$, as defined in chart $K_\text {A}$ by Eq. (31), transforms into its counterpart in $K_\text {B}$:

57 $$\begin{aligned} \hat{\Sigma }_2:=\left\{ (s, \eta ): \, |s-s_\text {A}| \leqslant \delta /\varepsilon , \eta = \eta _2 \right\} \,, \end{aligned}$$

where $s_\text {A}:=\sigma _\text {A}/\varepsilon$ and $\eta _2:= h_2/\varepsilon$. For a small enough ε, this transformation is well-defined and unambiguous. As a result, the point $\tilde{P}_{2,\varepsilon } \in \tilde{\Sigma }_2$ in chart $K_\text {A}$ corresponds to $\hat{P}_{2,\varepsilon } = (s_{\text {A},\varepsilon },\eta _2):= (\sigma _{\text {A},\varepsilon }/\varepsilon , h_2/\varepsilon ) \in \hat{\Sigma }_2$ in chart $K_\text {B}$.

#### Description of $\Pi _{2,3}^{(\varepsilon )}$

In chart $K_\text {B}$, the manifold $\hat{\mathcal {S}}_+$ and its perturbed counterpart $\hat{\mathcal {S}}_{+,\varepsilon }$ are strongly attractive, as obtained in Sect. 3.2.1. Starting from the point $\hat{P}_{2,\varepsilon }$, the trajectory rapidly descends along the fast fiber at $s=s_{\text {A},\varepsilon }$ until it approaches the manifold $\hat{\mathcal {S}}_{+,\varepsilon }$. Subsequently, it transitions to slow motion, drifting leftwards along $\hat{\mathcal {S}}_{+,\varepsilon }$, see Eq. (49), until it intersects the Poincaré section $\hat{\Sigma }_3$ at the point $\hat{P}_{3,\varepsilon } \in \hat{\Sigma }_3 \cap \hat{\mathcal {S}}_{+,\varepsilon }$.

#### Description of $\Pi _{3,4}^{(\varepsilon )}$

This transformation describes the passage through the fold point $F_\text {B}$, paralleling $\Pi _{1,2}^{(\varepsilon )}$; it occurs in chart $K_\text {B}$ and was characterized in Prop. 5. The trajectory starts from the point $\hat{P}_{3,\varepsilon } \in \hat{\Sigma }_3\cap \hat{\mathcal {S}}_{+,\varepsilon }$ and follows the manifold $\hat{\Gamma }_\varepsilon = \hat{\mathcal {S}}_{+,\varepsilon } \cup \hat{\mathcal {F}}_\varepsilon$. Initially, the trajectory moves along the slow branch $\hat{\mathcal {S}}_{+,\varepsilon }$, passing through an ε-neighborhood of the fold point $F_\text {B} = (s_\text {B},\eta _\text {B})$, Eq. (45). Near the fold point, the trajectory transitions to the fast fiber $\hat{\mathcal {F}}_\varepsilon$, then rapidly moving upwards to reach the point $\hat{P}_{4,\varepsilon }:= (s_{\text {B},\varepsilon }, \eta _4) \in \hat{\Sigma }_4$ at the Poincaré section $\hat{\Sigma }_4$ in Eq. (54). The fast fiber $\hat{\mathcal {F}}_0$ and its perturbed continuation $\hat{\mathcal {F}}_\varepsilon$ are transverse to $\hat{\Sigma }_4$, ensuring that the transition map $\Pi _{3,4}^{(\varepsilon )}$ is well-defined.

#### Description of $\Phi _{\text {B}, \text {A}}$

In analogy to $\Phi _{\text {A}, \text {B}}$, the transformation $\Phi _{\text {B}, \text {A}}: \hat{\Sigma }_4 \rightarrow \tilde{\Sigma }_4$ represents the switch from chart $K_\text {B}$ back to chart $K_\text {A}$. Recalling the relation between the coordinates, $\sigma = \varepsilon s$ and $\eta =h/\varepsilon$, the Poincaré section $\hat{\Sigma }_4$ defined in chart $K_\text {B}$ by Eq. (54) transforms in $K_\text {A}$ into:

58 $$\begin{aligned} \tilde{\Sigma }_4:=\left\{ (\sigma , h): \, |\sigma -\sigma _\text {B}| \leqslant \varepsilon \delta , h = h_4 \right\} \,, \end{aligned}$$

where $\sigma _\text {B}:= \varepsilon s_\text {B}$ and $h_4:= \varepsilon \eta _4$. For ε>0, this transformation is well-defined and unambiguous. As a result, the point $\hat{P}_{4,\varepsilon }:= (s_{\text {B},\varepsilon }, \eta _4) \in \tilde{\Sigma }_4$ in chart $K_\text {B}$ turns into the point $\tilde{P}_{4,\varepsilon } = (\sigma _{\text {B},\varepsilon }, h_4):= (\varepsilon s_{\text {B},\varepsilon }, \varepsilon \eta _4) \in \tilde{\Sigma }_4$ in chart $K_\text {A}$.

#### Description of $\Pi _{4,1}^{(\varepsilon )}$

In chart $K_\text {A}$, the manifold $\tilde{\mathcal {S}}_+$, and hence $\tilde{\mathcal {S}}_{+,\varepsilon }$, is strongly attractive, as we have found in Sect. 3.2. This means that starting from the point $\tilde{P}_{4,\varepsilon }$, the trajectory rapidly ascends along the fast fiber with $\sigma = \sigma _{\text {B}, \varepsilon }$ until it approaches the manifold $\tilde{\mathcal {S}}_{+,\varepsilon }$. After that, the trajectory proceeds to slow motion, moving rightwards along $\tilde{\mathcal {S}}_{+,\varepsilon }$ until it reaches the Poincaré section $\tilde{\Sigma }_1$ at the initial point $\tilde{P}_{1,\varepsilon } \in \tilde{\Sigma }_1 \cap \tilde{\mathcal {S}}_{+,\varepsilon }$, which closes the orbit.

Overall, starting at the intersection point $\tilde{P}_{1,\varepsilon }$ of the slow attracting manifold $\tilde{\mathcal {S}}_{+,\varepsilon }$ with the Poincaré section $\tilde{\Sigma }_1$, we have demonstrated that the trajectory completes one full loop, ultimately returning to the same point $\tilde{P}_{1,\varepsilon }$.

#### Numerical Results for the Perturbed Limit Cycle

**Fig. 8:**
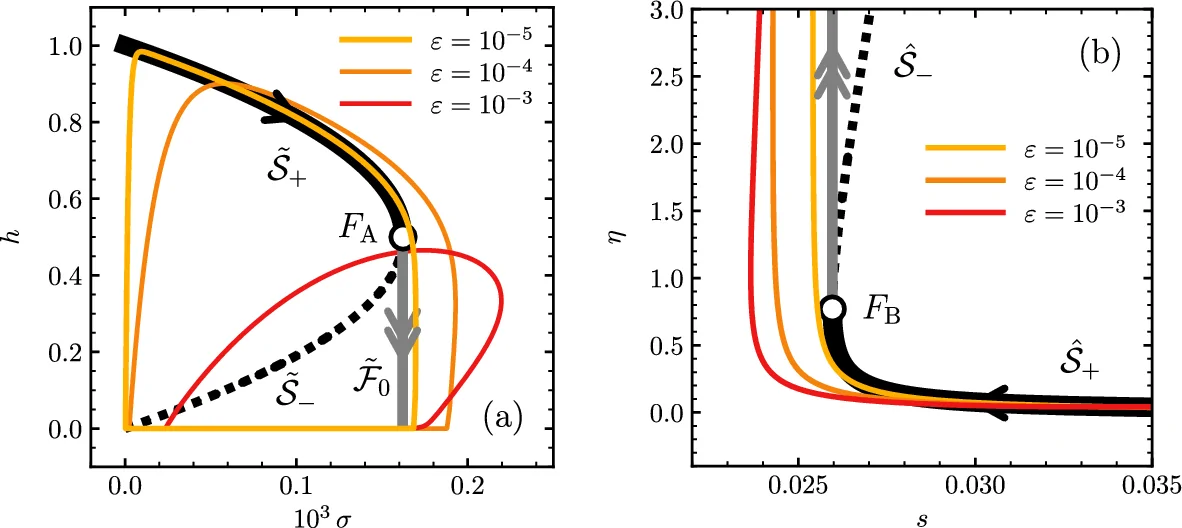
Numerical solutions of Eq. (9) for ε-perturbed limit cycle trajectories for three values of ε (solid colored lines) in comparison to the critical manifold (black lines) and fast fibers (gray lines); all other parameters are the same as in Fig. 1 and Table 3. The panels **a** and **b** correspond to the charts $K_\text {A}$ and $K_\text {B}$ in Fig. 7. **a** The full limit cycles in the coordinates (σ,h), together with the fold point $F_\text {A}$, the critical manifold $\tilde{\mathcal {S}}_\text {A}=\tilde{\mathcal {S}}_+ \cup \{ F_\text {A} \} \cup \tilde{\mathcal {S}}_-$ (black line, Eq. (23)), and the fast fiber $\tilde{\mathcal {F}}_0$ passing through $F_\text {A}$, cf. Sect 3.1. **b** The region near the fold point $F_\text {B}$ using the coordinates (s,η), showing parts of limit cycle trajectories (colored lines), the critical manifold $\hat{\mathcal {S}}_\text {B}=\hat{\mathcal {S}}_+ \cup \{ F_\text {B} \} \cup \hat{\mathcal {S}}_-$ (black line, Eq. (42)), and the fast fiber through $F_\text {B}$ (gray line), cf. Sect 3.2 (Color figure online)

After having constructed the limit cycle for arbitrarily small ε>0 as a perturbation of the critical manifold by means of GSPT, we test these predictions in Fig. 8 against a few representative limit cycle trajectories, interpolating between the real system and the idealised singular limit. The value $\varepsilon =10^{-3}$ corresponds to the experimental system (1) and decreasing ε increases the degree of timescale separation. Trajectories were obtained for $\varepsilon = 10^{-5}, 10^{-4}, 10^{-3}$ via numerical integration of Eq. (9) using Maple’s dsolve routine with the stiff solver option and an absolute accuracy of $10^{-10}$.

These numerical results neatly illustrate the emergence of fast–slow dynamics as ε decreases: the trajectories become increasingly aligned with the attractive branches of the slow critical manifold and the fast transition layers, as predicted by GSPT. In particular, the trajectory for $\varepsilon = 10^{-5}$ closely follows the ε-perturbed limit cycle, described in Sect. 3.3 and Fig. 7. The sharp directional changes near the fold points $F_\text {A}$ and $F_\text {B}$ correspond well to the fast, jump-like motions along the associated fast fibers, while the trajectory segments in between trace the slow manifolds with high accuracy. The trajectory for $\varepsilon = 10^{-4}$ shows already pronounced deviations from the critical manifold and is intermediate between the real system ($\varepsilon = 10^{-3}$) and the idealized one ($\varepsilon = 10^{-5}$), highlighting the gradual approach to the critical manifold and the role of ε in controlling the degree of timescale separation. The overall behavior of the numerical solutions not only corroborates the geometric picture established by the GSPT analysis, in Sects. 3.1 to 3.3, but also demonstrates how the limit cycle converges to its singular limit in a piecewise manner – composed of alternating slow drifts and fast transitions.

## Implications for the Chemical System

### Timescales and Asymptotic Estimates for the Period

One of the central practical questions related to rhythms concerns the period of oscillations and how it depends on the parameters of the system. Our GSPT analysis of the limit cycle reveals trajectory segments evolving on distinct timescales, which enable estimation of the oscillation period. In particular, we find two distinct branches of slow dynamics, evolving through the acidic domain and the other through the basic domain, see Sects. 3.1 and 3.2. These intervals of slow motion alternate with episodes of fast motion, forming a full cycle of oscillation, as shown in Sect. 3.3. Under sufficient timescale separation, the contribution of the fast segments to the total period of oscillation becomes negligible, and the oscillation period is well approximated by the time spent along the slow branches. It should be noted that in the singular limit ε=0, the period diverges and the limit cycle ceases to exist. However, for any arbitrarily small ε>0, a limit cycle emerges and its period can be well approximated by the time required to traverse the corresponding branches of the critical manifold.

We now estimate the characteristic times $\mathcal {T}_\text {acid}$ and $\mathcal {T}_\text {basic}$ associated with the slow motion along the two branches of the limit cycle. The time $\mathcal {T}_\text {acid}$ corresponds to motion along the perturbed slow manifold $\tilde{\mathcal {S}}_{+,\varepsilon }$ in the acidic domain, approaching the ε–vicinity of the fold point $F_\text {A}$. Similarly, $\mathcal {T}_\text {basic}$ describes the slow return through the basic domain along $\hat{\mathcal {S}}_{+,\varepsilon }$ toward the neighborhood of the neutral fold point $F_\text {B}$. Notably, as is inferred from Fig. 1d, the approach to $F_\text {B}$ is distinctly slower than that to $F_\text {A}$. It is therefore of particular interest to understand the origin of this asymmetry in the dynamics.

*Time spent in the acidic domain, along the branch* $\tilde{\mathcal {S}}_+$ *(chart* $K_\text {A}$ *).* Formally, the time $\mathcal {T}_\text {acid}$ for the motion along the acidic branch of the limit cycle is defined as

59 $$\begin{aligned} \mathcal {T}_\text {acid}(\varepsilon )&:= \int _{h_\text {start}}^{h_\text {end}} \left( \varepsilon \frac{d h_\varepsilon }{d \tau }\biggl |_{(\sigma ,h) \in \tilde{\mathcal {S}}_{+,\varepsilon }}\right) ^{-1} \textrm{d}h\,, \end{aligned}$$

where $d h_\varepsilon /d\tau$ is the ε-perturbed analog of the reduced dynamics in Eq. (27) along the manifold $\tilde{\mathcal {S}}_{+,\varepsilon }$. The integration over *h* starts at $h_\text {start}$ and ends at $h_\text {end}$, with the latter corresponding to the perturbed point $F_{\text {A},\varepsilon } = F_\text {A} + \mathcal {O}(\varepsilon ^{2/3})$, see Prop. 3. In a similar way, the starting value of integration $h_\text {start}$ is determined by the intersection of the fast fiber through $F_{\text {B},\varepsilon }$ with the slow manifold $\tilde{\mathcal {S}}_{+,\varepsilon }$.

Aiming to obtain $\mathcal {T}_\text {acid}$ to leading order in ε, we approximate the perturbed manifold and fold points by their singular counterparts, which lie within an $\mathcal {O}(\varepsilon ^{2/3})$ vicinity, as suggested by Propositions 3 and 5. Accordingly, the derivative in Eq. (59) is approximated by dh/dτ from Eq. (27), evaluated along the stable branch $\tilde{\mathcal {S}}_+$ of the critical manifold $\tilde{\mathcal {S}}_\text {A}$. The upper limit of integration is taken to be $h_\text {end} \approx h_\text {A}=1/2$, corresponding to the fold point $F_\text {A}$, see Eq. (24).

The critical manifold $\tilde{\mathcal {S}}_\text {A}$ is parametrized by the function φ(h) given by Eq. (23), and the lower integration boundary $h_\text {start}$ is determined by the condition $\varphi (h_\text {start}){\mathop {=}\limits ^{!}}\sigma _\text {B}$, where $\sigma _\text {B}= \varepsilon s_\text {B}$ with $s_\text {B}=\alpha (2\beta +1)K_h$ corresponding to the variable *s* taken at point $F_\text {B}$, see Eq. (45). This yields the quadratic equation $\alpha (\beta /C) K_h h_\text {start} (1-h_\text {start}) {\mathop {=}\limits ^{!}} \varepsilon \alpha (2\beta +1)K_h$ with respect to $h_\text {start}$. The larger root corresponds to the intersection with the stable part $\tilde{\mathcal {S}}_+$ of the critical manifold, and is given by $h_\text {start} = {1}/{2} + \sqrt{{1}/{4} - \varepsilon C (2 \beta +1)/\beta }$, which simplifies to $h_\text {start} = 1 + \mathcal {O}(\varepsilon )$ for small ε.

With these ingredients and Eq. (27), we obtain

60 $$\begin{aligned} \mathcal {T}_\text {acid}(\varepsilon )&\approx \int _1^{h_\text {A}} \left( \varepsilon \frac{d h}{d \tau }\biggl |_{(\sigma ,h) \in \tilde{\mathcal {S}}_+}\right) ^{-1} \textrm{d}h = -\frac{2\beta }{\varepsilon C} \int _1^{1/2} \frac{h-1/2}{h-h_*} \, \textrm{d}h = \frac{\beta }{\varepsilon C } w(h_*) \end{aligned}$$

with

61 $$\begin{aligned} w(h) = 1 - (1 - 2h) \ln \left( \frac{2 - 2h}{1 - 2h}\right) \,. \end{aligned}$$

Inserting $h_* \approx 0.0906$ from Eq. (6) into Eq. (60), we compute:

62 $$\begin{aligned} \mathcal {T}_\text {acid}(\varepsilon ) \approx 0.347 \, \frac{\beta }{\varepsilon C} \,. \end{aligned}$$

*Time spent in the basic domain, along the branch* $\hat{\mathcal {S}}_+$ *(chart* $K_\text {B}$ *).* As in the analysis of the acidic domain, we now evaluate the leading contribution to the oscillation period $\mathcal {T}_\text {basic}$ associated with motion along the branch of the limit cycle that lies in the basic domain. We approximate the perturbed manifold by the critical manifold $\hat{\mathcal {S}}_\text {B}$, whose stable branch $\hat{\mathcal {S}}_+$ is parametrized by the function $\psi (\eta )$, see Eq. (42). The time $\mathcal {T}_\text {basic}$ is obtained by integrating along η(t), in Eq. (50), as $\mathcal {T}_\text {basic}(\varepsilon ) \approx \int _{\eta _\text {start}}^{\eta _\text {end}} [ (d \eta / d t)|_{\hat{\mathcal {S}}_+} ]^{-1} \textrm{d}\eta$, where the integration limits $\eta _\text {start}$ and $\eta _\text {end}$ correspond to the intersections of the slow manifold $\hat{\mathcal {S}}_+$ with fast fibers passing through the fold points $F_\text {A}$ and $F_\text {B}$, respectively; see Fig. 7.

The starting point $\eta _\text {start}$ satisfies $\psi (\eta _\text {start}){\mathop {=}\limits ^{!}} s_\text {A}$, where $s_\text {A}=\sigma _\text {A}/\varepsilon$ (as in Eq. (57)) with $\sigma _\text {A} = \alpha \beta K_h/(4C)$ is a coordinate of the fold point $F_\text {A}$, see Eq. (24). This leads to the quadratic equation $\beta C/\eta _\text {start} + 1 + (\beta /C) \eta _\text {start} =\sigma _\text {A}/(\alpha K_h \varepsilon )$ in $\eta _\text {start}$, whose smaller root corresponds to the intersection with the stable branch of the critical manifold $\hat{\mathcal {S}}_+$, yielding $\eta _\text {start}$ close to zero. Hence, the term $\beta C/\eta _\text {start}$ dominates on the l.h.s. of the condition, and one finds $\eta _\text {start} = 4\varepsilon C^2 + \mathcal {O}(\varepsilon ^2)$, after also inserting $\sigma _\text {A}$. Alternatively, this results follows by Taylor expansion of the full expression, $\eta _\text {start} = [\beta -4\varepsilon C -\sqrt{(\beta -4\varepsilon C)^2-(8\varepsilon \beta C)^2}]/(8\varepsilon \beta )$. The end point of integration is approximated by $\eta _\text {end} \approx \eta _\text {B} = C$, corresponding to the fold point $F_\text {B}$.

Combining these results and using Eq. (50), the time it takes to traverse the basic branch is evaluated to

63 $$\begin{aligned} \mathcal {T}_\text {basic}(\varepsilon )&\approx \int _{4\varepsilon C^2}^{\eta _\text {B}} \left( \frac{d \eta }{d t}\biggl |_{\hat{\mathcal {S}}_+}\right) ^{-1} \textrm{d}\eta = \frac{\beta }{C h_*} \int _{4\varepsilon C^2}^{\eta _\text {B}} \frac{\eta _\text {B}^2-\eta ^2}{\eta ^2} \textrm{d}\eta \nonumber \\&= -\left. \frac{\beta }{h_*} \left( \frac{\eta }{C}+\frac{C}{\eta }\right) \right| _{4\varepsilon C^2}^{C} = \frac{\beta }{h_*} \frac{(1 - 4\varepsilon C)^2}{4\varepsilon C} \approx \frac{\beta }{4\varepsilon C h_* } \,. \end{aligned}$$

Inserting $h_* \approx 0.0906$ (Eq. (6)), we find

64 $$\begin{aligned} \mathcal {T}_\text {basic}(\varepsilon ) \approx 2.76 \, \frac{\beta }{\varepsilon C} \,. \end{aligned}$$

Thus, for the total oscillation period $\mathcal {T}(\varepsilon )$ to the leading order in ε, we obtain

65 $$\begin{aligned} \mathcal {T}(\varepsilon )&\approx \mathcal {T}_\text {acid}(\varepsilon ) + \mathcal {T}_\text {basic}(\varepsilon ) = \frac{\beta }{\varepsilon C} \left( \frac{1}{4 h_*} + w(h_*) \right) \approx 3.11 \frac{\beta }{\varepsilon C} \,. \end{aligned}$$

In the last step, we substituted $h_* \approx 0.0906$. Note that a close result with the prefactor 3.07 in the above expression follows if we approximate $w(h_*)\approx 1- \ln (2) + \mathcal {O}(h_*)$ for small $h_*$.

We observe that both characteristic times, $\mathcal {T}_\text {acid}$ and $\mathcal {T}_\text {basic}$, are primarily governed by the same parameter combination, $\beta / (\varepsilon C)$. As a result, both timescales—and thus the total oscillation period—increase as ε decreases. This behavior is qualitatively consistent with the fact that no limit cycle exists in the singular limit ε=0: the motion along the reduced manifold then requires infinite time. Geometrically, this corresponds to the expansion of the limit cycle as $\varepsilon \rightarrow 0$, with its right boundary (which, in the singular limit, coincides with the acidic fold point, $F_\text {A}$) shifting toward larger values of *s*. This trend is also evident in Figs. 4a or 8a, where one should recall that $\sigma = \varepsilon s$. Quantitatively, the scaling $\mathcal {T}_\text {acid}, \mathcal {T}_\text {basic} \sim \varepsilon ^{-1}$ is consistent with other GSPT results, such as those obtained for the Brusselator model (Engel and Méndez 2026).

The ratio between the two timescales is independent of ε and given by

66 $$\begin{aligned} \frac{\mathcal {T}_\text {acid}(\varepsilon )}{\mathcal {T}_\text {basic}(\varepsilon )} \approx 4h_*w(h_*) \,, \end{aligned}$$

where $w(h_*)$ is given by Eq. (61). This expression confirms that the motion along the upper branch is considerably faster, provided $h_* \ll 1$. For $h_* \approx 0.0906$ (see details following Eq. (6)), and thus the GSPT prediction yields $\mathcal {T}_\text {acid} / \mathcal {T}_\text {basic} \approx 0.126$. It is important to note, however, that this asymmetry between timescales arises independently of the smallness of ε.

*Timescales in physical units.* In dimensionless form, the durations $\mathcal {T}_\text {acid}$ and $\mathcal {T}_\text {basic}$ spent along the acidic and basic branches of the limit cycle are given by expressions Eqs. (60) and (63). To convert these to physical units, we use the rescaling relation $\varepsilon C = \varepsilon _1$ from Eq. (8) (see also Appendix A and Tables 2 and 3). This yields the dimensional timescales:

67a $$\begin{aligned} T_\text {acid}&= \frac{k_\text {M}}{v_\text {max}}\mathcal {T}_\text {acid} \approx \frac{k_\text {M}}{v_\text {max}} \frac{[\hbox {H+}_\text {ext}]}{k_\text {E1}} w(h_*) \,, \end{aligned}$$

67b $$\begin{aligned} T_\text {basic}&= \frac{k_\text {M}}{v_\text {max}}\mathcal {T}_\text {basic} \approx \frac{k_\text {M}}{v_\text {max}} \frac{[\hbox {H+}_\textrm{ext}]}{k_\textrm{E1} } \frac{1}{4h_*}\,, \end{aligned}$$

where $k_\text {M}$ and $v_\text {max}$ are the Michaelis–Menten constants. Their ratio $k_\text {max}:= v_\text {max}/k_\text {M}$ has the units of a rate ($\text {s}^{-1}$) and is the effective first-order urease turnover rate, defining the enzymatic timescale for conversion to physical units, see Sect. A. Furthermore, $k_\text {E1}$ is the enzymatic rate constant at low pH, the function *w*(*h*) is given by Eq. (61) and the fixed-point acid concentration of system Eq. (1) reads (cf. Eq. (6)):

68 $$\begin{aligned} h_* = \frac{[\hbox {H}^{+}_*]}{[\hbox {H}^{+}_\textrm{ext}]} = 1-2\frac{k_\text {S} [\hbox {S}_\textrm{ext}]}{k_\text {H} [\hbox {H}^{+}_\textrm{ext}]}\,. \end{aligned}$$

For sufficiently strong timescale separation, the total oscillation period is approximated by the sum

69 $$\begin{aligned} T&\approx T_\text {acid} + T_\text {basic} \nonumber \\&\approx \frac{k_\text {M}}{v_\text {max}} \frac{[\hbox {H}^{+}_\textrm{ext}]}{k_\textrm{E1} } \biggl (w(h_*) + \frac{1}{4h_*}\biggr )\,, \end{aligned}$$

with $h_*$ given in Eq. (68) and the function w(·) in Eq. (61). The magnitude of *T* is essentially set by the compound $k_\text {M} [\hbox {H}^{+}_\textrm{ext}]/(v_\text {max} k_\textrm{E1})$. For small values of $h_*$, as in our case with $h_* \approx 0.0906$, we find $T_\text {basic}/T_\text {acid} = (4 h_* w(h_*))^{-1} \approx 7.96$, see Eq. (66). Thus, the oscillation period is dominated by the slow excursion along the basic branch, so that $T \approx T_\text {basic}$ as given in Eq. (67b); this observation is qualitatively consistent with the numerical data presented in Fig. 1d. Generally, with the decrease in $h_*$, the oscillation period $T \approx T_\text {basic}$ grows as $h_*^{-1}$ and if $h_*$ approaches zero, where oscillations would disappear, the time spent on the lower branch diverges.

*Comparison of analytic predictions with numerical data.* Finally, we compare the asymptotic analytic predictions obtained in the limit of strong timescale separation with numerical results from direct integration of Eq. (9) for $\varepsilon = 10^{-5}, 10^{-4}, 10^{-3}$, using the parameter values listed in Table 3. The timescales spent along the slow branches in the acidic and basic domains, denoted by $\mathcal {T}_\text {acid}(\varepsilon )$ and $\mathcal {T}_\text {basic}(\varepsilon )$, are given analytically in Eqs. (60) and (63), respectively. As previously discussed, the total oscillation period excludes the fast transitions and is approximated by $\mathcal {T}(\varepsilon ) \approx \mathcal {T}_\text {acid}(\varepsilon ) + \mathcal {T}_\text {basic}(\varepsilon )$. From the numerical simulations, we estimate the corresponding timescales as the durations spent traveling from $F_\text {B}$ to $F_\text {A}$ and vice versa, denoted by $\tau _{\text {B}\rightarrow \text {A}}$ and $\tau _{\text {A}\rightarrow \text {B}}$, respectively. The full oscillation period is thus exactly $\tau = \tau _{\text {B}\rightarrow \text {A}} + \tau _{\text {A}\rightarrow \text {B}}$. The results, including the relative durations in each domain, are summarized in Table 1.

**Table 1:** Predictions and numerical results for the total oscillation period ($\mathcal {T}$ and $\mathcal {\tau }$, resp.) and the times spent on the two slow branches of the limit cycle, in the acidic and basic pH regimes. The analytical predictions $\mathcal {T}_\text {acid}$ and $\mathcal {T}_\text {basic}$ are given by Eqs. (60) and (63) with $\mathcal {T} = \mathcal {T}_\text {acid} + \mathcal {T}_\text {basic}$, for three different values of the scale separation, $\varepsilon =10^{-5}, 10^{-4}, 10^{-3}$. The data for $\mathcal {\tau }_{\text {B}\rightarrow \text {A}}$ and $\mathcal {\tau }_{\text {A}\rightarrow \text {B}}$ are taken from the numerical solutions of Eq. (9), shown in Fig. 8a; all other parameters are the same as in Fig. 1 and 3. The oscillation period $\tau = \tau _{\text {B}\rightarrow \text {A}} + \tau _{\text {A}\rightarrow \text {B}}$ consists of the time $\mathcal {\tau }_{\text {B}\rightarrow \text {A}}$ needed to move from points $F_\text {B}$ to $F_\text {A}$ and the time $\mathcal {\tau }_{\text {A}\rightarrow \text {B}}$ to close the loop. Values are rounded to three digits, and times are given in dimensionless form; physical times (in seconds) are obtained by dividing by $k_\text {max} \approx {6.17e-2\,\mathrm{\text {s}^{-1}}}$ (see Table 2)

ε	$\mathcal {T}$	$\mathcal {\tau }$	$\mathcal {T}_\text {acid}$	$\mathcal {\tau }_{\text {B}\rightarrow \text {A}}$	$\mathcal {T}_\text {basic}$	$\mathcal {\tau }_{\text {A}\rightarrow \text {B}}$	$\mathcal {T}_\text {basic}$/$\mathcal {T}_\text {acid}$	$\mathcal {\tau }_{\text {A}\rightarrow \text {B}}/\mathcal {\tau }_{\text {B}\rightarrow \text {A}}$
$10^{-5}$	8080	8460	901	975	7170	7480	7.96	7.67
$10^{-4}$	808	945	90.1	120	717	825	7.96	6.88
$10^{-3}$	80.8	90.2	9.01	23.0	71.7	67.2	7.96	2.92

For smaller values of ε ($\varepsilon = 10^{-5}, 10^{-4}$), the numerical results agree reasonably well with the theoretical predictions, with closer agreement for $\varepsilon = 10^{-5}$ and slightly larger deviations at $\varepsilon = 10^{-4}$. In both cases, the numerically estimated timescales $\tau _{\text {B}\rightarrow \text {A}}$ and $\tau _{\text {A}\rightarrow \text {B}}$ slightly overestimate the analytic values $\mathcal {T}_\text {acid}$ and $\mathcal {T}_\text {basic}$, respectively. The deviations are smaller for the basic segment. This discrepancy is expected because the analytic estimates capture only the slow drift along the critical manifold, whereas the numerical values include both slow and fast phases of motion. Consistent with theory, the ratio of time spent in the basic domain to that in the acidic domain remains nearly independent of ε, with the theoretical value being approximately 8. From the numerical data, we find this ratio to be 7.7 for $\varepsilon = 10^{-5}$ and 6.9 for $\varepsilon = 10^{-4}$.

For the original system [Eq. (1)], corresponding to $\varepsilon = 10^{-3}$, the agreement between numerical and analytic values is less accurate. The time $\tau _{\text {B}\rightarrow \text {A}} \approx 23$ overestimates the analytic value $\mathcal {T}_\text {acid} \approx 9$, while $\tau _{\text {A}\rightarrow \text {B}} \approx 67$ undershoots $\mathcal {T}_\text {basic} \approx 72$. As a result, the ratio $\tau _{\text {A}\rightarrow \text {B}} / \tau _{\text {B}\rightarrow \text {A}} \approx 3$ deviates considerably more from the theoretical prediction $\mathcal {T}_\text {basic} / \mathcal {T}_\text {acid} \approx 8$. We attribute these larger discrepancies to the fact that the shape of the limit cycle for $\varepsilon = 10^{-3}$ deviates visibly from the singular cycle, as can be seen in Fig. 8. Nevertheless, these effects cancel in the sum and our simple estimate $\mathcal {T}$ of the total oscillation period predicts its true value τ within 11%.

### Differential Transport Across the Membrane Required for Oscillations

Our study, including its geometric decomposition, not only provides a clear explanation for the existence and structure of the limit cycle but also uncovers additional insights into the conditions supporting oscillations. Among these, we identify a refined constraint for their emergence, expressed as $\alpha K_h > K_s$ (see Remark 1). In terms of the original parameters, this translates to

70 $$\begin{aligned} \frac{k_\text {H}}{k_\text {S}} > 2 \frac{[\hbox {S}_\textrm{ext}]}{[\hbox {H}^{+}_\textrm{ext}]}\,, \end{aligned}$$

where $k_\text {H}$, $k_\text {S}$ are transport rates across the membrane of the acid $\hbox {H}^{+}$ and substrate S, and $[\hbox {S}_\text {ext}]$, $[\hbox {H}^{+}_\text {ext}]$ are their external concentrations. Condition (70), which can be interpreted as a constraint on the differential transport, critical for sustained oscillatory behavior, sharpens requirements from prior studies Bánsági and Taylor (2014), such as ${k_\text {H}}/{k_\text {S}} > 1$, by explicitly linking transport rate asymmetry to external concentration ratios. Physically, this refined condition essentially imposes a condition on the relative strength of fluxes, namely: $k_\text {H} [\hbox {H}^{+}_\textrm{ext}] > 2 k_\text {S} [\hbox {S}_\textrm{ext}]$.

For the stability diagram plotted in the coordinates ($k_\text {H}/k_\text {S}$, $[\hbox {S}_\textrm{ext}]/[\hbox {H}^{+}_\textrm{ext}]$) – as in Fig. 2 and in Fig. 6 of  (Straube et al. 2023) – the condition in Eq. (70) defines the parameter domain above the line $k_\text {H}/k_\text {S} = 2 [\hbox {S}_\textrm{ext}]/[\hbox {H}^{+}_\textrm{ext}]$ (which corresponds to $K_h/K_s = \alpha ^{-1}$ in Fig. 2). This inequality thus sets a lower bound for the domain of oscillations in the present reduced model, as oscillations can occur only in the parameter region above this line, where differential transport across the membrane is sufficiently strong. This analytical prediction aligns well with the numerically determined oscillatory domain reported in  (Straube et al. 2023) (cf. their Fig. 6). Notably, the numerical data from that study further suggest that Eq. (70) must be satisfied together with the condition $[\hbox {S}_\text {ext}] \gtrapprox [\hbox {H}^{+}_\text {ext}]$ to support sustained oscillations.

## Conclusion

In this work, we have uncovered the geometric structure underlying the oscillatory dynamics of a biochemical pH oscillator, namely, the urea–urease reaction network confined to a lipid vesicle (Bánsági and Taylor 2014; Muzika et al. 2019; Miele et al. 2022). Our analysis has built on a simple yet realistic model in two variables (Straube et al. 2023) and has used geometric singular perturbation theory (GSPT) as the central mathematical tool, providing us with a natural framework for resolving the fast–slow structure of the emerging limit cycle. One result of our study is a closed formula, Eq. (69), for estimating the oscillation period of the experimental reaction network. For relevant parameter values, the obtained deviation of 11% from the actual period (see Table 1) lies within a practically meaningful range, given the complexity of the underlying chemical system. Yet, establishing general bounds on the prediction error remains as a demanding task.

Despite the inherent presence of distinct fast and slow processes, the model lacks a single dominant small parameter that would directly control the timescale separation. Instead, it is controlled by several small dimensionless parameter combinations, none of which suffices to characterize the system’s multiscale behavior. We have overcome this issue by a suitable formal coupling of such combinations, which enables the application of classical GSPT techniques.

Using this framework, we have identified distinct critical manifolds corresponding to the slow segments of the limit cycle—one in the acidic region, the other in the basic region. To resolve their local structures, we have introduced two separate rescalings, each tailored to the respective pH domain. This approach enables a detailed analysis of the system near the associated fold points, where normal hyperbolicity is lost. In particular, we have proven that these points are generic folds rather than canards, contrary to an earlier speculation (Straube et al. 2021). Using GSPT extended to degenerate equilibria (Krupa and Szmolyan 2001), we have rigorously analyzed the dynamics through these non-hyperbolic regions. Finally, we have assembled a global picture of the oscillatory mechanism by matching the local analyses through suitably constructed transition maps.

In our study, we have focused on the main structure of the fast–slow limit cycle, yielding a first approximation within a simple coherent framework in terms of a single small parameter ε. Going beyond this, one may refrain from coupling the parameter combinations and work directly with two small parameters $\varepsilon _1$ and $\varepsilon _2$. This would induce the analysis of various regimes, where one of the parameters is small but fixed and the other is sent to 0. Note that this may uncover even more subtle substructures, as demonstrated for the peroxidase–oxidase reaction via a multiscale analysis of the Olsen model (Kuehn and Szmolyan 2015). One could then also analyze a double-singular limit when $\varepsilon _1, \varepsilon _2 \rightarrow 0$, see also  (Kuehn et al. 2022).

The present work treats $\varepsilon _1$ and $\varepsilon _2$ as the primary singular perturbation parameters, which arguably constitutes the most natural asymptotic regime associated with the underlying chemical system. Table 3 shows that the dimensionless parameter combinations $\varepsilon _1$ and $\varepsilon _2$ are considerably smaller than all other dimensionless parameters in terms of the experimentally relevant parameter values; in particular, we have considered $\beta =\mathcal {O}(1)$ as fixed. However, one could perform a simultaneous limit of more than two small parameters and, for example, also regard β as small and consider limits of the form $\beta = \mathcal {O}(\varepsilon ^{1/2})$ or $\mathcal {O}(\varepsilon )$, extending Eq. (8). Our preliminary analysis of such scenarios shows that the choice $\beta = \mathcal {O}(\varepsilon ^{1/2})$ yields a similar overall geometric picture as elaborated above, after choosing suitable rescalings of the (*s*, *h*) plane. However, taking $\beta = C_\beta \varepsilon$ with $C_\beta = 20$, such that the original value of β is restored for $\varepsilon =10^{-3}$, and sending $\beta , \varepsilon _1, \varepsilon _2$ to zero simultaneously, upon $\varepsilon \rightarrow 0$, results in a similar dynamics and only marginally perturbed limit cycles, compared to the original set of parameter values. Under this scaling of β, using charts (*s*, *h*) instead of (σ,h) and $(s, \varepsilon ^{-2}h)$ instead of (s,η) resolves the fold points $F_\text {A}$ and $F_\text {B}$, respectively, analogously as in Fig. 8. The further analysis of this distinguished limit of the present dynamical system appears to be promising, in particular, with respect to an improved prediction of the oscillation period.

Eventually, there is a strong motivation to extend the present analysis of the deterministic dynamics to stochastic regimes. In biochemical systems, molecular fluctuations are inevitable and become increasingly pronounced as the system size decreases. Intrinsic noise, arising from the discreteness and finite copy numbers of molecules, is known to modify the dynamics of monostable enzymatic reaction networks (Thomas et al. 2010, 2012), and can even lead to noise-induced oscillations (Thomas et al. 2013). In oscillatory systems, such noise effects can become especially pronounced near singularities such as fold points, where the system’s sensitivity to perturbations increases (Engel and Olicón-Méndez 2024). Indeed, an earlier numerical study by some of us (Straube et al. 2021) within the framework of the chemical master equation (Winkelmann and Schütte 2020) demonstrated that periodic oscillations become increasingly irregular as noise intensifies, due to a decrease of vesicle size, eventually leading to a breakdown of rhythmicity. This suggests that the geometric structure identified through GSPT in the deterministic setting may continue to shape the dynamics as long as the noise is moderate. Supporting this idea, prior theoretical work has shown that slow manifolds persist in a probabilistic sense, and deviations due to noise can be quantified precisely (Berglund and Gentz 2006).

## Data Availability

No datasets were generated. All numerical results can be reproduced from the equations and parameters fully specified in the manuscript.

## Connection with a Realistic Model of the Urea–Urease Reaction

In the following, we the relate the simplified model Eq. (1) and the model derived in Straube et al. (2023), which is an already reduced, yet reliable model of the full urea–urease reaction network (Bánsági and Taylor 2014).

Let $[\hbox {S}]$ and $[\hbox {H}^{+}]$ denote the time-dependent concentrations substrate molecules (urea) and hydrogen ions within the vesicle and $[\hbox {S}_\text {ext}]$ and $[\hbox {H}^{+}_\text {ext}]$ refer to their constant values in the vesicle’s exterior, which acts as a reservoir. In terms of the dimensionless variables $s=[\hbox {S}]/[\hbox {S}_\text {ext}]$ and $h=[\hbox {H}^{+}]/[\hbox {H}^{+}_\text {ext}]$, the reaction kinetics of the urease-catalyzed hydrolysis of urea was shown to obey the dynamical system (Straube et al. 2023)

A1a $$\begin{aligned} \frac{ds}{dt}&= -k_\textrm{cat}(s,h) \, s + k_\textrm{S}\, (1-s)\,, \end{aligned}$$

A1b $$\begin{aligned} \frac{dh}{dt}&= -k \,p(s,h) \,h + k_\textrm{H}\,(1-h)\,, \end{aligned}$$

for rate constants $k_\textrm{S},k_\textrm{H},k>0$. The effective rate $k_\textrm{cat}(s,h) > 0$ expresses Michaelis–Menten kinetics with a pH-dependent reaction velocity and has the product form

A2 $$\begin{aligned} k_\textrm{cat}(s,h)&= k_\textrm{cat}^\textrm{M}(s [\hbox {S}_\text {ext}]) \cdot f_\textrm{H}(h[\hbox {H}^{+}_\text {ext}]) \end{aligned}$$

with

A3 $$\begin{aligned} k_\textrm{cat}^\textrm{M}([\hbox {S}]) = \frac{v_\textrm{max} }{ k_\textrm{M}+[\hbox {S}]} \quad \text {and} \quad f_\textrm{H}([\hbox {H}^{+}]) = \frac{1}{1 + [\hbox {H}^{+}]/k_\textrm{E1} + k_\textrm{E2} / [\hbox {H}^{+}]} \,, \end{aligned}$$

where $v_\text {max}$ is the maximum reaction speed, $k_\text {M}$ the Michaelis–Menten constant, and $k_\text {E1}$ and $k_\text {E2}$ are enzyme-specific $\hbox {H}^{+}$ concentrations. The bell-shaped function $f_\textrm{H}$ attains its maximum at $[\hbox {H}^{+}]_\text {max} = (k_\text {E1} k_\text {E2})^{1/2}$ and its width scales as the ratio $\beta ^{-2}:=k_\text {E1} / k_\text {E2}$. The function p(s,h)⩾0 appearing in Eq. (A1b) reads

A4 $$\begin{aligned} p(s,h)&= -\frac{b(h)}{2} + \frac{1}{2}\sqrt{b(h)^2+4c(s,h)}\, \end{aligned}$$

with the abbreviations

A5a $$\begin{aligned} b(h)&:= 1 + k' [\hbox {H}^{+}_\text {ext}]h + ( 1 - h^{-1} ) \, k_\textrm{H} /k \,, \end{aligned}$$

A5b $$\begin{aligned} c(s,h)&:= 2 \,k_\textrm{cat}(s,h) k' [\hbox {S}_\text {ext}] s / k \ge 0\,, \end{aligned}$$

and a constant $k^{\prime }>0$.

For the parameters of the model, Eqs. (A1)–(A5), we use the same values as in Straube et al. (2023), which are listed in Table 2. This choice of parameters introduces a timescale separation into the model, which we exploit in this work to develop the GSPT analysis. Additionally, it allows for some *a priori* simplifications of the functional dependencies.

**Table 2:** Parameter values of the real-world chemical system for substance concentrations, enzyme properties, reaction rates, rates of differential transport across the vesicle membrane, and derived quantities

Parameter	Symbol	Value
External urea concentration	$[\hbox {S}_\textrm{ext}]$	${3.8 \times 10^{-4}}$ M
External proton concentration	$[\hbox {H}^{+}_\textrm{ext}]$	${1.3 \times 10^{-4}}$ M
Maximum reaction speed	$v_\textrm{max}$	${1.85 \times 10^{-4}}{\textrm{M}}\, \textrm{s}^{-1}$
Michaelis-Menten constant	$k_\textrm{M}$	${3 \times 10^{-3}}$ M
Enzyme constant (low pH)	$k_\textrm{E1}$	${5 \times 10^{-6}}$ M
Enzyme constant (high pH)	$k_\textrm{E2}$	${2 \times 10^{-9}}$ M
Ammonia protonation rate	$k_2$	${4.3 \times 10^{10}}{\textrm{M}}^{-1}\,{\textrm{s}}^{-1}$
Ammonium deprotonation rate	$k_{2r}$	${2.4 \times 10^{1}}\,\textrm{s}^{-1}$
Proton transport rate	$k_\textrm{H}$	${9 \times 10^{-3}}\,\textrm{s}^{-1}$
Urea transport rate	$k_\textrm{S}$	${1.4 \times 10^{-3}}\,\textrm{s}^{-1}$
Ammonia outflow rate	*k*	$=k_\text {S}$
Ammonium ion outflow rate	$k_{+}$	$=k_\text {S}$
$$k_2 / (k_{2r}+k_+) = $$	$k^{\prime }$	1.79e9 $$\textrm{M}^{-1}$$
$v_\text {max} / k_\text {M} =$	$k_\text {max}$	${6.17 \times 10^{-2}}\,\textrm{s}^{-1}$

**Table 3:** Dimensionless parameter combinations of model (1)

$K_h$	$k_\text {H}/k_\text {max}$	0.15
$K_s$	$k_\text {S}/k_\text {max}$	0.023
*K*	$k/k_\text {max}$	0.023
α	$[\hbox {H}^{+}_\textrm{ext}] \big / (2[\hbox {S}_\textrm{ext}])$	0.17
β	$\sqrt{k_\textrm{E2} / k_\textrm{E1}}$	0.02
$\varepsilon _1$	$\sqrt{k_\textrm{E1} k_\textrm{E2}} \, \big / \, [\hbox {H}^{+}_\textrm{ext}]$	${7.7 \times 10^{-4}}$
$\varepsilon _2$	$\alpha / (k'[\hbox {H}^{+}_\textrm{ext}])$	${7.3\times 10^{-7}}$

To obtain the simplified model of the main text, Eq. (1) together with Eqs. (2)–(4), we start by noting that Eqs. (A1a) and (A1b) imply bounded solutions h(t),s(t)∈[0,1]. This follows from inspection of the signs of the right hand side of both equations and provided that the initial conditions satisfy these bounds. Moreover, the numerical solution for the limit cycle ( (Straube et al. 2023) and Fig. 1) indicates that s≲0.25. Hence, there is a scale separation $s \ll k_\text {M} / [\hbox {S}_\text {ext}] \approx 7.9$ or, equivalently, for the substrate concentration, $[\hbox {S}] \ll k_\text {M}$. It allows us to approximately treat the Michaelis–Menten kinetics as a first-order (linear) reaction and to replace the catalytic rate by a constant, $k_\textrm{cat}^\textrm{M}([\hbox {S}]) \approx v_\text {max} / k_\text {M} =: k_\text {max}$, which has units of inverse time and represents the effective enzyme turnover rate setting the intrinsic catalytic timescale. Additionally, the substrate concentration, over large parts of the limit cycle (cf. Fig. 1), is much smaller inside of the vesicle than in its exterior, s≪1 or $[\hbox {S}] \ll [\hbox {S}_\text {ext}]$, which suggests approximating the last term in Eq. (A1a) by a constant, $k_\textrm{S}(1-s) \approx k_\textrm{S}$, thereby simplifying the present analysis. Regarding the function *b*(*h*), we note that $k' [\hbox {H}^{+}_\text {ext}] \approx {2.3\times 10^5}$ is a large constant and thus $1 + k' [\hbox {H}^{+}_\text {ext}] h\approx k' [\hbox {H}^{+}_\text {ext}]h$, except for tiny values of *h*, namely, for very low proton concentration $[\hbox {H}^{+}] \lesssim 1/k'$ in the basic regime, $\textrm{pH} \gtrsim 9.3$, which is not reached by the limit cycle (Fig. 1d). This motivates the approximation of *b*(*h*) as given in Eq. (A5a) by

A6 $$\begin{aligned} b^{\text {app}}(h) := k' [\hbox {H}^{+}_\text {ext}]h + ( 1 - h^{-1} ) \, k_\textrm{H} /k \approx b(h) \,. \end{aligned}$$

In summary, provided that the solutions obey $0 \leqslant s \ll \min (k_\text {M} / [\hbox {S}_\text {ext}], 1)$ and $(k' [\hbox {H}^{+}_\text {ext}])^{-1} \ll h \leqslant 1$, Eq. (A1) can be replaced by

A7a $$\begin{aligned} \frac{ds}{dt}&= - k_\textrm{max} f_\textrm{H}(h[\hbox {H}^{+}_\text {ext}]) \, s + k_\textrm{S} \,, \end{aligned}$$

A7b $$\begin{aligned} \frac{dh}{dt}&= -k \,p(s,h) \,h + k_\textrm{H}\,(1-h)\,. \end{aligned}$$

These equations coincide with the dynamical system Eq. (1) written in terms of the rescaled time $t' = k_\textrm{max} t$ (and subsequently omitting the prime) and upon introducing dimensionless parameters $K_s:= k_\textrm{S}/k_\textrm{max}$, $K_h:= k_\textrm{H}/k_\textrm{max}$, and $K:= k/k_\textrm{max}$ and the functions given in Eqs. (2)–(4):

A8a $$\begin{aligned} r(h) := f_\textrm{H}(h[\hbox {H}^{+}_\text {ext}]) \,, \qquad q(s,h) := K p(s,h) h \,, \end{aligned}$$

and, using the approximation Eq. (A6),

A8b $$\begin{aligned} v(h) := K b^{\text {app}}(h) h = \alpha K \varepsilon _2^{-1} h^2 -K_h(1-h) \,, \end{aligned}$$

with parameters

A9a $$\begin{aligned} \beta&:= \sqrt{ k_\text {E2} / k_\text {E1}},&\alpha&:= ~ [\hbox {H}^{+}_\textrm{ext}] / (2 [\hbox {S}_\textrm{ext}] ) \,, \end{aligned}$$

A9b $$\begin{aligned} \varepsilon _1&:= \sqrt{k_\text {E1} k_\text {E2}} \big / [\hbox {H}^{+}_\text {ext}],&\varepsilon _2&:= 1/ (2 k' [\hbox {S}_\textrm{ext}] ) \,. \end{aligned}$$

We note that, in chemistry, one often uses logarithmic variables (pS, pH) instead of (*s*, *h*), which are related to each other through

A10a $$\begin{aligned} \begin{aligned} \hbox {pS}&:= -\log _{10}([\hbox {S}] / 1\,\textrm{M}) &  = -\log _{10}(s) - \log _{10}([\hbox {S}_\textrm{ext}] / 1\,\textrm{M}) , \end{aligned}\end{aligned}$$

A10b $$\begin{aligned} \begin{aligned} \hbox {pH}&:= -\log _{10}([\hbox {H}^{+}] / 1\,\textrm{M}) &  = -\log _{10}(h) - \log _{10}([\hbox {H}^{+}_\textrm{ext}] / 1\,\textrm{M}) \,. \end{aligned} \end{aligned}$$

Table 3 lists the values of the above parameter combinations, for which the reduced and simplified system (1) exhibits a limit cycle, shown in Fig. 1. Previous work (Straube et al. 2023) and Fig. 2 show that the existence of a limit cycle sensitively depends on the ratio α of the external concentrations, which we keep fixed here. From Table 3, one identifies $$\varepsilon _1$$ as a small parameter of the system; this ratio specifies by how much the external pH value deviates from the point, where the catalytic reaction velocity $$\propto f_\textrm{H}$$ attains its maximum, which is at $$\hbox {pH}=7$$. Another small parameter is $$\varepsilon _2$$, which is inversely proportional to the large parameter combination $$k' [\hbox {H}^{+}_\text {ext}] = \alpha /\varepsilon _2$$ mentioned above. The latter is the ratio between the $$\hbox {H}^{+}$$ concentration in the exterior of the vesicle and the effective equilibrium constant $1/k^{\prime }$ of the $${\hbox {NH}_{3} + \hbox {H}^{+} \rightleftharpoons \hbox {NH}_4^+}$$ conversion reaction, including the small outflow of $${\hbox {NH}_4+}$$ across the membrane. For the values given in Table 3, one finds that $$\varepsilon _1^2$$ and $$\varepsilon _2$$ are of similar order of magnitude: $$\varepsilon _1^2 / \varepsilon _2 \approx 0.81.$$

## A Primer on the Analysis of Fold Points in GSPT

Here, we summarize the standard conditions that characterize *fold points* and distinguish *generic folds* (Kuehn 2015). Consider the slow time formulation of a fast–slow system,

$$ \dot{x} = f(x,y), \quad \varepsilon \dot{y} = g(x,y) \quad (0 < \varepsilon \ll 1)\,, $$

where *x* and *y* denote the slow and fast variables, respectively, and ε is a small parameter separating their timescales. A point $P:=(x_0,y_0)$ on the critical manifold

$$\mathcal {C}:= \{ (x,y): g(x,y)=0 \}$$

is called a *generic fold point* if the following conditions hold:

1. *Fold point conditions:*
  $$\begin{aligned} g(P) = 0, \quad \partial _y g(P) = 0\,, \end{aligned}$$
  which imply that *P* lies on the critical manifold $\mathcal {C}$ and that the fast subsystem has a singularity at *P*. In particular, $\partial _y g(P) = 0$ corresponds to the loss of normal hyperbolicity of $\mathcal {C}$ at *P*, since the linearization of the fast subsystem degenerates.
2. *Nondegeneracy conditions:*
  $$\begin{aligned} \partial _y^2\,g(P) \ne 0, \quad \partial _x g(P) \ne 0\,, \end{aligned}$$
  where $\partial _y^2 g(P) \ne 0$ ensures that the fold is quadratic (and therefore nondegenerate) in the fast variable, and $\partial _x g(P) \ne 0$ guarantees that the critical manifold varies regularly with the slow variable, preventing degeneracies also in the reduced (slow) flow.
3. *Transversality condition:*
  $$\begin{aligned} f(P) \ne 0\,, \end{aligned}$$
  which ensures that the slow flow crosses the fold transversally, allowing trajectories to pass between attracting and repelling branches of the critical manifold. Together, conditions (i)-–(iii) characterize *P* as a *generic fold point*, a structurally stable and nondegenerate singularity of the critical manifold where normal hyperbolicity is lost in a controlled manner. Away from fold points, where $\partial _y g \ne 0$, the critical manifold is normally hyperbolic.

If any of the conditions (i)–-(iii) fails, the point *P* is a *nongeneric fold point*. In particular, if the transversality condition (iii) is violated so that f(P)=0 then *P* is called a *folded singularity*. Such points represent a degeneracy where the slow flow is tangent to the fold, and this tangency is a necessary (though generally not sufficient) condition for the existence of *canard* solutions (Benoît et al. 1997, Krupa and Szmolyan 2001, Kuehn 2015)—special trajectories that follow both attracting and repelling branches of the critical manifold for significant time intervals.

## Footnotes

1 For notational simplicity, we omit the subscript *A* for the subsets $\tilde{\mathcal {S}}_{\pm }\subset \tilde{\mathcal {S}}_A$.

2 For notational simplicity, we omit the subscript *B* for the subsets $\hat{\mathcal {S}}_{\pm }\subset \hat{\mathcal {S}}_B$.
